# Monosodium Glutamate Perturbs Human Trophoblast Invasion and Differentiation through a Reactive Oxygen Species-Mediated Pathway: An In-Vitro Assessment

**DOI:** 10.3390/antiox12030634

**Published:** 2023-03-03

**Authors:** Indrani Mukherjee, Subhrajit Biswas, Sunil Singh, Joyeeta Talukdar, Mohammed S. Alqahtani, Mohamed Abbas, Tapas Chandra Nag, Asit Ranjan Mridha, Surabhi Gupta, Jai Bhagwan Sharma, Supriya Kumari, Ruby Dhar, Subhradip Karmakar

**Affiliations:** 1 Department of Biochemistry, All India Institute of Medical Sciences, New Delhi 110029, India; 2 Amity Institute of Biotechnology (AIB), Amity University, Noida 201301, India; 3 Amity Institute of Molecular Medicine & Stem Cell Research (AIMMSCR), Amity University, Noida 201301, India; 4 Radiological Sciences Department, College of Applied Medical Sciences, King Khalid University, Abha 61421, Saudi Arabia; 5 BioImaging Unit, Space Research Centre, Michael Atiyah Building, University of Leicester, Leicester LE1 7RH, UK; 6 Electrical Engineering Department, College of Engineering, King Khalid University, Abha 61421, Saudi Arabia; 7 Electronics and Communications Department, College of Engineering, Delta University for Science and Technology, Gamesa 35712, Egypt; 8 Department of Anatomy, All India Institute of Medical Sciences, New Delhi 110029, India; 9 Department of Pathology, All India Institute of Medical Sciences, New Delhi 110029, India; 10 Department of Reproductive Biology, All India Institute of Medical Sciences, New Delhi 110029, India; 11 Department of Obstetrics & Gynaecology, All India Institute of Medical Sciences, New Delhi 110029, India

**Keywords:** monosodium glutamate, reactive oxygen species, oxidative stress, trophoblast, invasion, differentiation, endoplasmic reticulum stress

## Abstract

The overproduction of reactive oxygen species (ROS) has been associated with various human diseases. ROS exert a multitude of biological effects with both physiological and pathological consequences. Monosodium glutamate (MSG), a sodium salt of the natural amino acid glutamate, is a flavor-enhancing food additive, which is widely used in Asian cuisine and is an ingredient that brings out the “umami” meat flavor. MSG consumption in rats is associated with ROS generation. Owing to its consumption as part of the fast-food culture and concerns about its possible effects on pregnancy, we aimed to study the impact of MSG on placental trophoblast cells. MSG exposure influenced trophoblast invasion and differentiation, two of the most critical functions during placentation through enhanced production of ROS. Similar findings were also observed on MSG-treated placental explants, as confirmed by elevated Nrf2 levels. Ultrastructural studies revealed signs of subcellular injury by MSG exposure. Mechanistically, MSG-induced oxidative stress with endoplasmic reticulum stress pathways involving Xbp1s and IRE1α was observed. The effect of MSG through an increased ROS production indicates that its long-term exposure might have adverse health effect by compromising key trophoblast functions.

## 1. Introduction

Reactive oxygen species (ROS) refer to oxygen-containing molecules having an unpaired electron or to unstable compounds such as hydrogen peroxide (H_2_O_2_), superoxide (O^2−^), hydroxyl (OH^−^), and peroxyl radicals [1]. ROS are naturally produced, the majority being a by-product of mitochondrial oxidative phosphorylation [2]. At physiological levels, ROS are produced at the preimplantation milieu and are found to be critical for early embryogenesis. However, the redox balance in the early window of embryo development needs to be precisely controlled owing to the detrimental effects by the overproduction of ROS [3]. Any deviation from redox homeostasis can have serious consequences affecting the embryo implantation and its subsequent development.

In pregnancy-related disorders, such as preeclampsia (PE) and pregnancy-induced hypertension, an increase in oxidative stress is considered to be a major inducing factor [4]. Oxidative stress (OS) is reported to cause tissue damage and other related pathologies [5,6,7,8]. In PE patients, ROS increases the level of lipid peroxidation, mainly malondialdehyde (MDA), which is majorly owing to the decline in activity of the antioxidant defense machinery [9]. Monosodium glutamate (MSG) is a known food additive and is used as a major ingredient in most commercially prepared foods [10]. The global size of the glutamic acid market has been estimated to be over 2.9 million tons in 2014 and more than 4 million tons by 2023 [11]. The MSG-related revenue is expected to be worth more than USD 15.5 billion by 2023, growing with an estimated compound annual growth rate above 7.5% up to 2023 [11]. With such an enormous appetite for this additive in the food industry, the health concerns are also substantial given that MSG administration increases the number of pachytene stage cells in primary spermatocytes [12,13] and induces OS [14,15] and free radical generation [16,17]. In addition, obesity [18,19,20,21], disorders of the central nervous system [10,22,23], hepatic damage [24,25,26], reproductive malfunctions [12,27,28,29], cardiovascular diseases [30,31], and hypertension [32,33,34] are considered to be some of the major side effects of MSG.

However, the detailed molecular pathogenesis regarding MSG consumption is poorly understood. The placenta is important in sustaining a pregnancy and supporting fetal growth and development. Trophoblast cells constitute the functional and structural components of the placenta to support pregnancy by orchestrating delicate feto-maternal crosstalk and its endocrine function. This involves the exchange of nutrients and waste materials across the feto-placental unit. Trophoblast cells are transiently invasive [35,36] and participate in the remodeling of the maternal uterine matrix and its vasculature to gain access to its nutrient-rich milieu. Successful pregnancy depends on the efficiency of this process, which depends on an efficient trophoblast differentiation program.

Therefore, in the present study, we investigated the effect of MSG on placental trophoblast cells and extrapolated its possible consequences on placental function. Whether maternal consumption of MSG will result in its trans-placental transport to the developing embryo is still debatable. However prolonged consumption and exposure could affect the function of placental trophoblast cells either directly or indirectly through dysregulating the redox homeostasis at the feto-placental interface.

## 2. Materials and Methods

### 2.1. Cell Culture

HTR-8/SVneo cells and BeWo choriocarcinoma cells were obtained from American Type Culture Collection (ATCC, Rockville, MD, USA). Cells from relatively early passages (R = 3) were cultured using RPMI-1640 (HyClone) containing 1% penicillin-streptomycin (Invitrogen) and 10% fetal bovine serum (FBS) (Invitrogen) [37].

Cells (1 × 10^6^) were plated in a 90-mm petri dish and cultured at 37 °C within a humidified chamber with 5% CO_2_. The experiments were broadly divided into acute and chronic stimulation. Acute stimulation groups were divided into three sub-groups: (i) control, (ii) cells treated with 25 mM MSG for 24 h, and (iii) cells treated with 50 mM MSG for 24 h (Figure S1A,B). Chronic stimulation groups were divided into two sub-groups: (i) control and (ii) cells treated with 25 mM MSG every alternate day for 16 days (Figure S1C,D).

To confirm the induction of OS in acute conditions, cells were (iv) treated with 10 mM N-acetyl cysteine (NAC), a potent antioxidant, for 24 h and (v) pretreated with NAC for 2 h followed by MSG treatment for 24 h. To perform rescue experiments in chronic conditions, cells were (a) treated with 10 mM NAC on every alternate day and (b) pretreated with NAC for 2 h followed by 25 mM MSG treatment on every alternate day, for 16 days. At 50–60% confluence, cells were kept in a phenol red-free medium containing 5% FBS and treated with NAC and MSG.

### 2.2. Explant Culture

First-trimester human placental samples of different gestational ages between 8–10 weeks were obtained from medically terminated pregnancies (n = 10) after delivery, and their tissue explants were cultured.

Small tissue sections (10 mg) were cut and plated in a collagen-I-coated single well of 12-well plates (Corning). Tissue sections were washed thoroughly using 1× phosphate-buffered saline (PBS), and RPMI-1640 with 10% FBS and 1% penicillin–streptomycin was added. Tissues were cultured at 37 °C within a humidified chamber under 5% CO_2_ [37]. For experimental purposes, early villi sections were divided into two groups: (i) control and (ii) sections treated with 50 mM MSG for 24 h (Supplementary Figure S1E). Tissues were treated with TriZol (Thermo Fisher Scientific, Washington, DC, USA) for RNA isolation. The explants were divided into two more sub-groups: (i) explants treated with 10 mM NAC for 24 h and (ii) explants pretreated with NAC for 2 h followed by 50 mM MSG treatment for 24 h to assess ROS generation. The explants were stimulated for 72 h also to study their ultrastructural changes after chronic stimulation by MSG.

### 2.3. RNA Isolation and Reverse Transcription–Quantitative Polymerase Chain Reaction (RT-qPCR)

Total RNA was extracted from cells using an RNA Simple Total RNA isolation kit (Promega RNA Tissue/Cell Miniprep System) according to the manufacturer’s instructions. In-column DNase digestion was performed to obtain DNase-free RNA. RNA was quantified using Nanodrop (Thermo Fisher Scientific, Inc., Washington, DC, USA), and agarose gel electrophoresis was performed to assess RNA integrity. RNA samples were then reverse transcribed into cDNA using a Verso cDNA synthesis kit (AB1453A, Thermo Fisher Scientific, Inc., Washington, DC, USA). RT-qPCR was performed using a DyNAmo Flash SYBR Green qPCR kit (F415S, Thermo Fisher Scientific, Inc., Washington, DC, USA). The reaction cycles were as follows: incubation at 95 °C for 7 min, 40 cycles at 95 °C for 15 s and 60 °C for 20 s. The PCR products were subjected to a melting curve analysis to confirm amplification specificity. mRNA levels were then normalized with respect to mRNA levels of GAPDH using the 2^−ΔΔCq^ method [37]. PCR primer sequences are provided in Table S1.

### 2.4. Cell Viability and Proliferation Assay

Cells were plated in 96-well plates and treated with different doses of MSG (5, 25, 50, 100, 150, and 200 mM) for 24 h. Water-soluble tetrazolium-1 (WST-1) assay was performed to assess the proliferation and viability of cells following the manufacturer’s protocol. Results were analyzed by comparison with the standard curve [37].

### 2.5. Cell Cycle and Apoptosis

For cell cycle analysis, MSG-treated trophoblast cells (HTR-8/SVneo and BeWo) were collected from the log phase, washed with 1× PBS, and fixed with 70% ethanol overnight at 4 °C. After washing twice with 1× PBS, cells were resuspended and incubated in 500 µL PBS containing 100 µg/mL RNase and 50 µg/mL propidium iodide (PI) for 30 min at room temperature. Cells were analyzed by flow cytometry (ThermoFisher Scientific, Cat No. V13242, Washington, DC, USA), and the percentage of cell proliferation was analyzed using GraphPad Prism v.6.01.

The extent of apoptosis was analyzed by flow cytometry using Annexin V-FITC/propidium iodide (PI) double-label assay, according to the manufacturer’s protocol (BMS500FI/100CE, Invitrogen). Cells (1 × 10^6^) were trypsinized and incubated in 100 μL binding buffer containing 5 μL AnnexinV/FITC and 10 μL 20 μg/mL PI for 15 min in the dark at room temperature. A total of 10,000 events/runs were acquired, and the results were analyzed using FlowJo software (Ashland, OR, USA) [37].

### 2.6. ROS Production

Cells were plated in 12-well plates and treated with 25 and 50 mM MSG for 24 h (acute group) and 25 mM MSG every alternate day for 16 days (chronic group). Then, 5 μM CellRox (Cellular ROS Assay Kit, ab186029S) was added to each well and was incubated for 30 min at 37 °C. For the acute group, cells were pretreated with 10 mM NAC for 2 h followed by MSG treatment for another 24 h. Cells were then washed thrice with 1× PBS and analyzed by flow cytometry (BD FACSCanto) [37].

For assaying thiobarbituric acid reactive substances (TBARS), cells were harvested after acute or chronic treatment as previously described. This assay was also performed with the following early placental explants groups: control, 50 mM MSG treatment, 10 mM NAC, and pre-treatment with NAC for 2 h followed by 50 mM MSG treatment. Thiobarbituric Acid Reaction Species Assay (Cayman Chemicals) was performed to estimate the levels of MDA, the final product of lipid peroxidation. TBARS concentrations were measured at 532 nm.

### 2.7. Western Blotting

Cells were lysed using a radioimmunoprecipitation assay buffer, containing protease and phosphatase inhibitors. Proteins (60 μg) were separated using 10% discontinuous sodium dodecyl sulfate–polyacrylamide gel electrophoresis and transferred to a polyvinylidene difluoride membrane. The membrane was blocked with 5% non-fat milk for 1 h and then incubated with primary antibodies at 1:1000 dilution overnight at 4 °C. The membrane was washed in Tris-buffered saline containing 0.1% Tween 20 and then incubated with horseradish peroxidase (HRP)-conjugated secondary antibodies at room temperature for 1 h. Protein bands were visualized by a densitometer using an enhanced chemiluminescence (ECL) detection kit (NCI4106; Thermo Fisher Scientific, Inc.) [37]. The same membrane was used for re-probing with an anti-GAPDH antibody (Cell Signaling Technology, Inc., Beverly, MA, USA) at 1:1500 dilution. Band intensities were estimated using Image J, and the expression of proteins was normalized with respect to that of GAPDH.

Antibodies used have been listed in Table 1.

### 2.8. Gelatin Zymography

Bioactivities of secreted matrix metalloproteinases (MMPs)–2/9 in the conditioned media of HTR-8/SVneo cells were measured using substrate gel gelatin zymography. Proteins were separated using an 8% polyacrylamide gel containing 0.1% gelatin. The gel was then washed with 2.5% Triton X-100 solution for 1 h to renature proteins, washed further with distilled water, and incubated in activation buffer (150 mM NaCl, 10 mM CaCl_2_, 50 mM Tris, and 0.025% sodium azide) at 37 °C with gentle shaking overnight. An amount of 0.1% Coomassie brilliant blue (0.1%) was used to stain the gel for 30 min. The gel was destained using 10% acetic acid solution and visualized [37].

### 2.9. Wound Healing Assay/Cell Migration Assay

Cells were seeded in 6-well plates at a density of 2 × 10^5^ cells per well and incubated to attain 80–90% confluence. A small pipette tip was used to generate a scratch through the cell monolayers, and the debris was removed using 1× PBS. After treating the cells for 24 h, as previously mentioned, images were acquired at regular intervals until complete wound healing (filling up of the scratch with cells), using a phase-contrast microscope [37].

### 2.10. Matrigel Invasion Assay

The invasion assay was performed using a 24-well insert system (8 μm pores; Transwell chamber, Merck Millipore, Darmstadt, Germany). The surface of the insert plate was coated with 50 μL diluted Matrigel (400 µg/mL) (356234; Becton Dickinson and Company, Franklin Lakes, NJ, USA, 1:9 in RPMI-1640). An aliquot of 200 uL of HTR-8/SVneo cell suspension (1 × 10^5^ cells) was seeded into the insert, and FBS-supplemented RPMI was added to the reservoir well. Cells were treated and cultured for 24 h. After incubation, a swab was used to remove the remaining cells from the upper insert. Trophoblast cells that infiltrated and reached the other side of the insert were fixed using ice-cold methanol and stained with 4′,6-diamidine-2′-phenylindole dihydrochloride (D9564, Sigma-Aldrich) for nuclear staining and Alexa Fluor 488 Phalloidin (green) (Cell Signalling Technology, Beverly, MA, USA) for cytoplasmic staining. Images obtained were captured using an upright microscope (TI-E, 601869, Nikon Laser Scanning Confocal Microscope) at 200× magnification [37].

### 2.11. Transmission Electron Microscopy (TEM)

Early placental explant tissues (1 mm^3^/piece) and cells pellets (1 × 10^6^) treated with 50 mM MSG for 72 h were fixed with Karnovsky’s fixative at 4 °C, rinsed with 0.1 M phosphate buffer (pH 7.4) twice, and post-fixed with 1% osmium tetroxide for 1 h. Samples were then dehydrated in acetone, embedded in Araldite CY212, and polymerized. Sections (60–70 nm thick) were cut and stained with uranyl acetate and lead citrate and then examined using a Talos 200S transmission electron microscope (Thermo Scientific, Inc., Washington, DC, USA) [37].

### 2.12. Immunohistochemistry

Early placental explant tissues treated with 50 mM MSG for 72 h were washed in PBS and fixed overnight in 4% buffered formalin at 4 °C. Samples were then washed to remove the fixative, and paraffin-embedded 5 µm-thick sections were cut using a microtome (36,000; ThermoFisher Scientific, Washington, DC, USA). The sections were deparaffinized, rehydrated, and microwaved for 20 min in Tris-EDTA buffer (pH 9.0) for antigen retrieval. Endogenous peroxidase was quenched by treating the sections with 3% H_2_O_2_ for 15 min. Nonspecific binding was blocked using a BlockAid Blocking Solution (Invitrogen). The sections were incubated with rabbit anti-Nrf2 antibody (dilution: 1:200; sc-365949, Santa Cruz Biotechnology, Inc., Dallas, TX, USA) and rabbit anti-Xbp1s antibody (dilution: 1:200; 40435, Cell Signal Technology, Beverly, MA, USA) at 4 °C in a humid chamber for 12–14 h. Subsequently, the sections were incubated with fluorophore-conjugated anti-rabbit secondary antibody (Dako, Santa Clara, CA, USA) at room temperature for i h, washed thrice with 0.1 M TBS, and mounted with DPX mounting medium (Merck, Millipore, Germany). The sections were observed using a compound microscope [37]. Scoring was done on the basis of intensity (i) and percentage of immunopositivity of the cells (Pi). The i-values were assigned on a scale of 0–3, where 0, 1, 2, and 3 indicated no, weak, moderate, and strong staining. The P value varied from 0–100%, and the final score was derived from the sum of (i × P) value as shown in the equation below. This score ranges from 0–300 [38].

H-score = (0 × P0) + (1 × P1) + (2 × P2) + (3 × P3)(1)

For nuclear factor erythroid 2-related factor 2 (Nrf2):

H score of the control explant = (0 × 5) + (1 × 5) + (2 × 10) + (3 × 80) = 265

H score of the MSG treated explant = (0 × 5) + (1 × 75) + (2 × 15) + (3 × 5) = 120

For X-box binding protein 1 (Xbp1s):

H score of the Control explant = (0 × 7) + (1 × 80) + (2 × 10) + (3 × 3) = 109

H score of the MSG treated explant = (0 × 5) + (1 × 8) + (2 × 85) + (3 × 2) = 184

H-score was calculated using mean ± standard deviation (SD).

### 2.13. Haematoxylin and Eosin Staining

Slides containing tissue sections were placed in a staining jar and deparaffinized by submerging into three series of absolute xylene for 4 min, followed by treatment with 100%, 100%, 95%, 90%, and 70% ethanol for 4 min each. The slides were then washed with running tap water for 2 min, submerged into Harris hematoxylin (Sigma-Aldrich, Darmstadt, Germany) for 2 min, washed with running tap water for 2 min, and finally mounted with a coverslip using DPX mounting agent after dehydration in graded ethanol. Explant tissue sections were stained with hematoxylin and eosin to study placental villi, syncytial knot formation, and stromal pathology.

### 2.14. Collagen Special Staining (a Modified Masson’s Trichrome Staining)

Slides containing early explant tissues were placed in a staining jar, deparaffinized by submerging into three series of absolute xylene, and rehydrated through a graded series of ethanol. The slides were then submerged in warm Bouin’s solution (Sigma-Aldrich, Darmstadt, Germany) at 60 °C for 45 min and then thoroughly washed with running tap water until the yellow color in the samples disappeared. To differentiate nuclei, the slides were then immersed in modified Weigert’s hematoxylin for 8 min and then washed with running water for 2 min. To stain cytoplasm and erythrocytes, the slides were submerged in anionic dyes and acid fuchsin (Sigma-Aldrich, Darmstadt, Germany) for 5 min and washed with running tap water for 2 min. Next, the slides were treated with phosphomolybdic acid solution (a mordant) for 10 min and immediately submerged into methyl blue (C.I. 42780, Merck, Germany) solution for 5 min to stain collagen and fibroblasts. Then, the slides were thoroughly washed with running tap water for 2 min and treated with 1% acetic acid solution for 1 min. The slides were then dehydrated through a graded series of ethanol, dipped into absolute xylene for 1 min, mounted with a coverslip using DPX mounting reagent, and observed using a microscope (TI-E, 601869, Nikon Laser Scanning Confocal Microscope) at 200× magnification.

### 2.15. Enzyme-Linked Immunosorbent Assay (ELISA)

ELISA was performed to assess the levels of β subunit of human chorionic gonadotropin (*β-hCG*) in conditioned media of BeWo cells and placental explant cultures using a human hCG-beta ELISA Kit (EH235RB, Thermo Fisher Scientific, Inc., Washington, DC, USA). Conditioned media (100 µL) was added to each well of a 96-well plate and incubated for 2.5 h at room temperature with gentle shaking. Then the media was removed, and the plate was washed four times. Then, 100 µL biotin conjugate was added to each well and incubated for 1 h at room temperature with gentle shaking, and the plate was washed again. Then, 100 µL streptavidin-HRP solution was added to each well and incubated for 45 min at room temperature with gentle shaking. The plate was washed again, and 100 µL of 3,3′,5,5′-tetramethylbenzidine substrate was added to each well and incubated for 30 min at room temperature in the dark with gentle shaking. Stop solution (50 μL) was added to each well and gently mixed until the color changed from blue to yellow [37]. The absorbance was read at 450 nm within 30 min.

### 2.16. Statistical Analysis

All experiments were repeated at least thrice. Data are presented as mean ± standard deviation (SD). The statistical significance was assessed by Student’s ***t***-test using GraphPad Prism v5.0 (San Diego, CA, USA), with a *p* value < 0.05 considered statistically significant.

## 3. Results

### 3.1. Effect of MSG Stimulation on Viability and Proliferation of Trophoblast Cells

**Figure 1:**
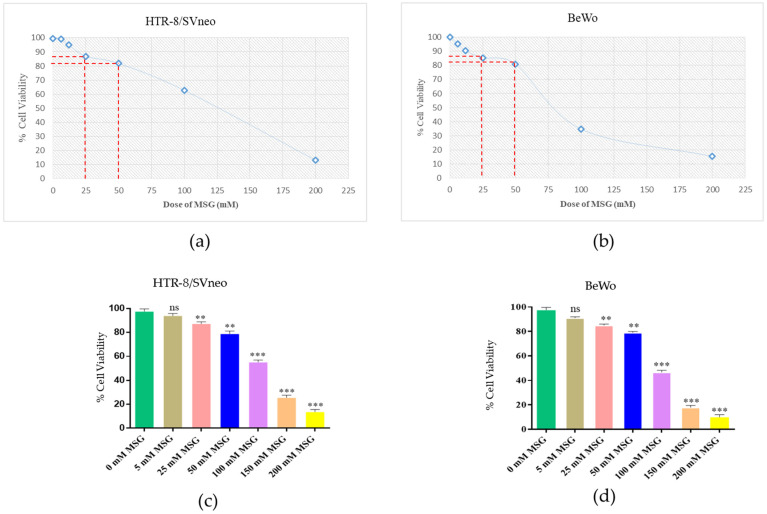
Effect of MSG on the viability of BeWo and HTR-8/SVneo cells. WST-1 assay and trypan blue staining were performed to assess and quantify cell viability (**a**,**b**) The dotted red line depicts the chosen experimental doses of MSG; the trophoblast cells HTR-8/SVneo and BeWo were also stained with trypan blue after treatment with MSG, and the results were plotted graphically (**c**,**d**). Results are representative of at least three independent experiments. ** *p <* 0.01; *** *p <* 0.001; ns (non-significant).

The sub-lethal dose of MSG and its toxic effects on trophoblast cell lines were evaluated using WST-1 assay. A dose-dependent reduction in viability was observed in HTR-8/SVneo (Figure 1a) and BeWo (Figure 1b) cells. Trypan blue staining (Figure 1c,d) showed similar results. Annexin-V/PI staining showed that more than 75% cells were viable when treated with 25 and 50 mM MSG for acute stimulation and 25 mM MSG for chronic stimulation in HTR-8/SVneo and BeWo cells. (Figure S2a–d) Cell cycle analysis following acute stimulation (24 h) with 25 and 50 mM MSG showed a nonsignificant change in the G1 and S phases in BeWo cells, as compared to those of the control group. A significant increase in proliferation in the G2 phase was noticed in BeWo cell stimulated with 50 mM MSG (Figure S2e); however, no significant change was observed in the percentage of HTR-8/SVneo cells in the S phase compared to that of untreated cells. (Figure S2f). Moreover, no significant difference in cell proliferation was observed between the control and the treated groups in BeWo (Figure S2g–j) and HTR-8/SVneo (Figure S2k–n) cells. Therefore, both 25 and 50 mM MSG doses were used for studying the acute effects of MSG, and a 25 mM dose was used for chronic stimulation studies.

### 3.2. Acute Stimulation with MSG Increases Differentiation and Invasion in Trophoblast Cells and Early Placental Explants

**Figure 2:**
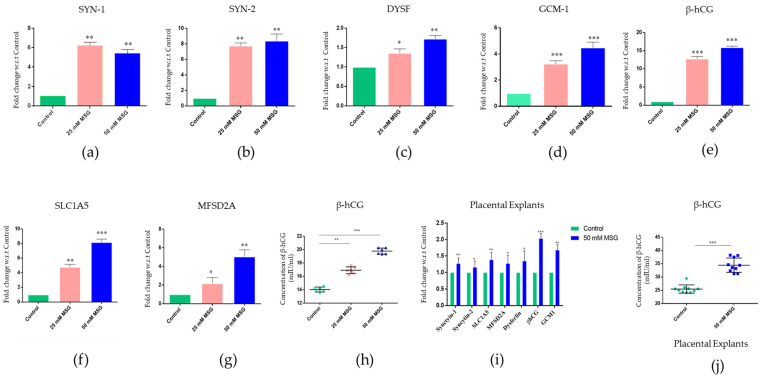
Expression of differentiation markers after acute stimulation of MSG in the trophoblast cells and placental explant tissues. qPCR showing fold change in the mRNA expression of differentiation markers in BeWo cells after acute stimulation of MSG (25 and 50 mM) for 24 h. The results were analyzed by the 2^−ΔΔC.T.^ method (ΔC.T. = C.T. value of sample −ΔC.T. value of internal reference gene) (**a**–**g**); quantification of *β-hCG* in conditioned media of the treated early placental explants using ELISA (**h**); qPCR showing fold change in mRNA expression of differentiation markers in tissue explants from early placentas treated with 50 mM MSG (**i**); quantification of *β-hCG* levels in the conditioned media of treated early placental explants using ELISA. (n = 10) (**j**). All data represent mean ± standard deviation. Results are representative of at least three independent experiments. * *p <* 0.05; ** *p <* 0.01; *** *p <* 0.001.

To assess the effect of MSG on trophoblast differentiation, BeWo cells were used to analyze the expression of trophoblast differentiation markers including syncytin-1 (SYN-1), syncytin-2 (SYN-2), and glial cells missing-1 (GCM1), dysferlin (DYSF), β subunit of human chorionic gonadotropin (*β-hCG*), solute carrier family 1 Member 5 (SLC1A5), and major facilitator superfamily domain containing 2A (MFSD2A). MSG (25 and 50 mM) significantly increased the mRNA levels of *SYN-1, SYN-2, DYSF, GCM-1, β-hCG, SLC1A5,* and *MFSD2A* (Figure 2a–g). *β-hCG* concentration was also evaluated in the conditioned media of cells, and significant upregulation of *β-hCG* level was observed after acute stimulation with MSG (Figure 2h). We next evaluated the effect of MSG in the early placental explants derived from first trimester chorionic villi obtained from medically terminated pregnancy cases (8–12 weeks of pregnancy). The expression of *SYN-1, SYN-2, DYSF, GCM1,* and *β-hCG* was significantly upregulated (*p* < 0.01). mRNA levels of SLC1A5 and MFSD2A, the receptors of SYN-1 and SYN-2, respectively, showed similar trends (Figure 2i). *β-hCG* protein levels were significantly upregulated in early explants treated with 50 mM MSG (Figure 2j). Therefore, MSG significantly upregulated trophoblast differentiation markers in trophoblast cells and tissue explants.

**Figure 3:**
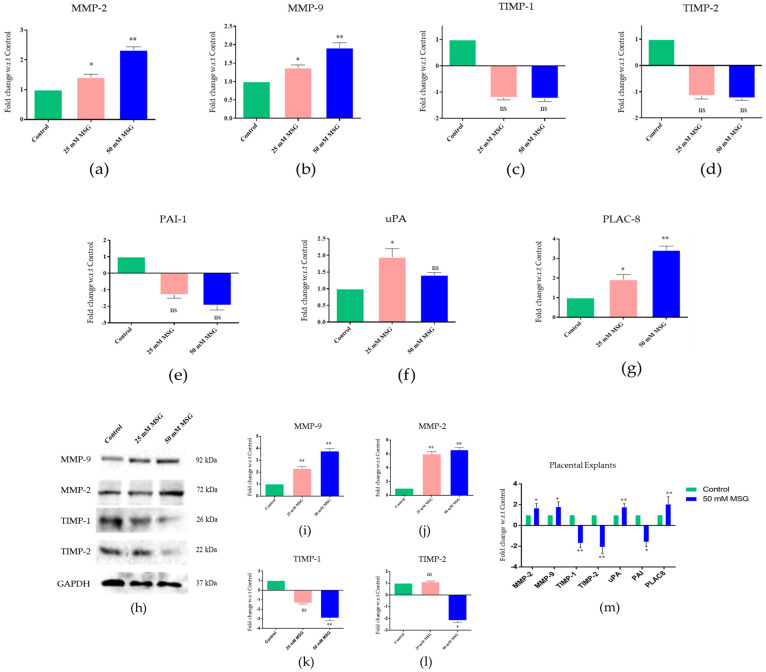
MSG alters the level of proteases and their inhibitors. qPCR showing the changes in mRNA expression of *MMP*−*2*, *MMP*−*9*, *TIMP*−*1*, *TIMP*−*2*, *UPA*, *PAI*, and *PLAC8* in HTR-8/SVneo cells. The results were analyzed by the 2^−ΔΔC.T^. method (ΔC.T. = C.T. value of sample −ΔC.T. value of internal reference gene) (**a**–**g**); Western blot showing the levels of MMPs and their inhibitors (**h**); graphical representation of band intensities of proteins quantified using ImageJ. Original blots are presented in Figure S4a–e (**i**–**l**); mRNA expression of *MMP*−*2*, *MMP*−*9*, *TIMP*−*1*, *TIMP*−*2*, *UPA*, *PAI*, and *PLAC8* in early placental explants treated with 50 mM MSG (n = 10) (**m**). All data in the figure represent mean ± standard deviation. Results are representative of at least three independent experiments. * *p <* 0.05; ** *p <* 0.01; ns (non-significant).

We next analyzed the effects of MSG on trophoblast invasion. Using the extravillous trophoblast cells HTR-8/SVneo, we assessed the mRNA expression of invasion-associated genes *MMP-2*, *MMP-9*, and *uPA*, and their inhibitors *TIMP-1, TIMP-2,* and *PAI-1*, respectively. Significant upregulation of mRNA expression of *MMP-2*, *MMP-9*, and *uPA* was observed with MSG treatment compared to those of the untreated controls (*p* < 0.05). (Figure 3a,b,e). MSG treatment (50 mM) showed an insignificant decrease in the mRNA expression of *TIMP-2* and *PAI* without altering the mRNA expression of *TIMP-1* (Figure 3c,d,f). Interestingly, we observed an elevated mRNA expression of *ONZIN*/placenta associated 8 (*PLAC8*) with both doses of MSG (*p* < 0.01) (Figure 3g). *ONZIN* has been earlier reported [39] to be positively associated with the invasiveness and migratory behavior of trophoblast cells. The Western blot analysis showed similar trends, with MSG stimulation increasing the expression of MMPs and decreasing the expression of TIMPs. However, TIMP-1 expression was reduced by 50 mM MSG (Figure 3h–l).

**Figure 4:**
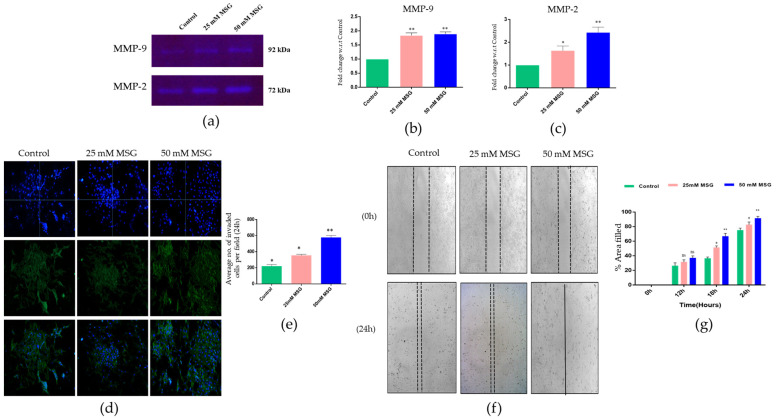
Gelatin zymography showing the activity of MMP-2 and MMP-9. Band intensities were quantified using ImageJ. Original gels are presented in Figure S5 (**a**–**c**); matrigel invasion assay representing the average number of invaded cells in four random fields as analyzed using ImageJ and graphical plot of the data (n = 3) (**d**,**e**); wound-healing assay showing fold difference in the extent of the area filled by cells as represented graphically for each time interval (**f**,**g**). All data in the figure represent mean ± standard deviation. Results are representative of at least three independent experiments. * *p <* 0.05; ** *p <* 0.01; ns (non-significant).

To further confirm that the effect of MSG on the pro-invasiveness of HTR-8/SVneo cells, as seen in the invasion assay, is owing to the secretion of MMPs, a functional assay was performed using gelatin zymography. Results showed a significant upregulation of MMP-2 and MMP-9 activities in MSG-stimulated HTR-8/SVneo cells (Figure 4a–c). Matrigel invasion assay showed a similar trend with a significant upregulation of the average number of invading HTR-8/SVneo cells per field after the acute stimulation of MSG (Figure 4d,e). In the wound-healing assay, HTR-8/SVneo cells showed a significantly higher migration rate than did the control cells, following MSG treatment (*p* < 0.01) (Figure 4f,g). To further elucidate the effect of short-term MSG exposure on early placental explants, we assessed the gene expression of the invasion markers. mRNA expression of *MMP-2*, *MMP-9*, and *uPA* was significantly upregulated in the placental explants; however, those of *TIMP-1* and *TIMP-2* were significantly downregulated (Figure 3m). mRNA expression of *PLAC8* was significantly higher in the MSG-treated group. Therefore, acute stimulation of MSG is responsible for elevated differentiation and invasion potentials in trophoblast cells and early placental explants.

### 3.3. Long-Term Chronic Stimulation of MSG Decreases Differentiation and Invasion of Trophoblasts

**Figure 5:**
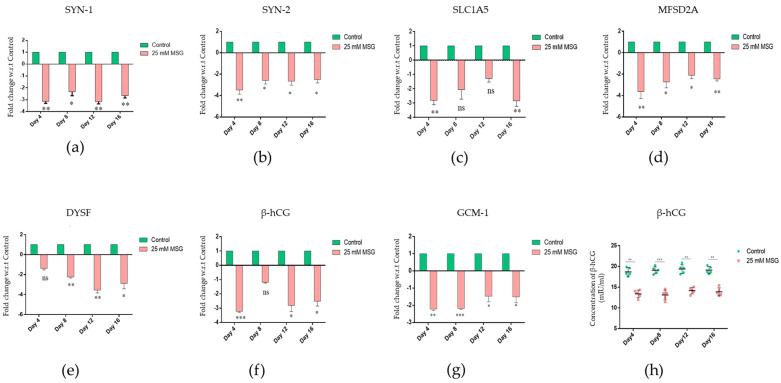
Expression of differentiation markers after chronic stimulation of MSG in BeWo cells. qPCR showing fold change in mRNA expression of differentiation markers after chronic stimulation of MSG (25 mM) for 16 consecutive days. The results were analyzed by the 2^−ΔΔC.T^ method (ΔC.T. = C.T. value of sample −ΔC.T. value of internal reference gene) (**a**–**g**); quantification of *β-hCG* level in conditioned media of treated cells using ELISA (**h**). All data are shown as mean ± standard deviation. Results are representative of at least three independent experiments. * *p <* 0.05; ** *p <* 0.01; *** *p <* 0.001. ns (non-significant).

Next, we assessed mRNA expression of differentiation markers in BeWo cells on Day 4, Day 8, Day 12, and Day 16 after chronic stimulation (16 days) by 25 mM MSG by treating cells every alternate day for the entire span of 16 days. Surprisingly, we observed an opposite trend with a significant decrease in the expression of *SYN-1, SYN-2, SLC1A5, MFSD2A, DYSF, GCM1*, and *β-hCG* (*p* < 0.01) (Figure 5a–g). We assessed the expression from conditioned media on days 4, 8, 12, and 16, which showed a significant reduction (Figure 5h).

**Figure 6:**
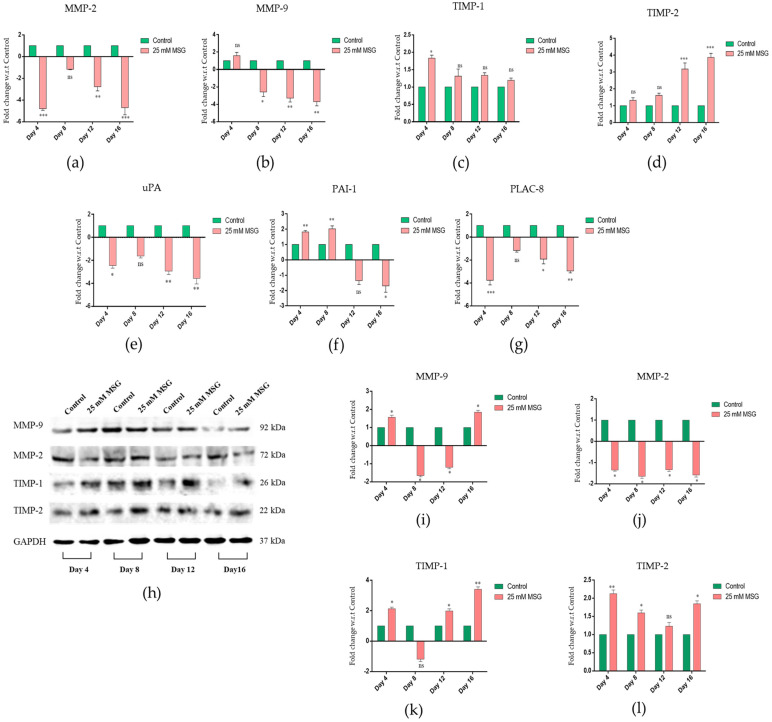
Effects of chronic stimulation of MSG on invasive and migratory properties of HTR-8/SVneo cells. qPCR showing the change in mRNA expression *MMP-2, MMP-9, TIMP-1, TIMP-2, UPA, PAI,* and *PLAC-8*, in cells treated with 25 mM MSG for 16 consecutive days. The results were analyzed by the 2^−ΔΔC.T^ method (ΔC.T. = C.T. value of sample −ΔC.T. value of internal reference gene) (**a**–**g**); Western blots showing the expression MMPs and TIMPs (**h**); graphical representation of the band intensities normalized with respect to GAPDH expression. Original blots are presented in Figure S6a–e (**i**–**l**). All data are shown as mean ± standard deviation. Results are representative of at least three independent experiments. * *p <* 0.05; ** *p <* 0.01; *** *p <* 0.001. ns (non-significant).

To determine the effect of chronic stimulation of MSG on the invasion of trophoblast cells, we analyzed the expression of genes previously studied during acute stimulation. Interestingly, we observed that the expression of *MMP-2, MMP-9*, and *uPA* were significantly downregulated on days 4, 12, and 16. *MMP-9* expression was downregulated by approximately three-fold, and *MMP-2* and *uPA* showed similar trend (though not statistically significant on Day 8 for *MMP-2* and *uPA*, and a nonsignificant increase in *MMP-9* on Day 4) (Figure 6a,b,e). mRNA expression of *TIMP-1* and *TIMP-2* were upregulated on all four days. The fold changes for *TIMP-1* on days 8, 12, and 16 and *TIMP-2* on days 4 and 8 were not significant (Figure 6c,d). In contrast, *PAI-1* level was slightly upregulated on days 4 and 8 and was eventually downregulated on Day 16 of stimulation (Figure 6f). *PLAC8* level was significantly downregulated on days 4, 12, and 16 (Figure 6g).

**Figure 7:**
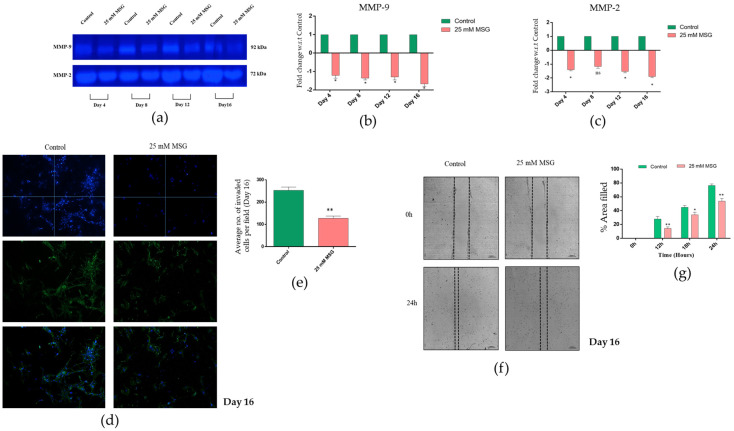
Gelatin zymography of cell supernatant showing the activities of MMP−2 and MMP−9 on days 4, 8, 12, and 16 of MSG stimulation. Band intensities were quantified using ImageJ and graphically plotted. Original gels are presented in Figure S7 (**a**–**c**); matrigel invasion assay showing the effect of chronic MSG exposure on the invasiveness of cells and graphical representation of the data (**d**,**e**); scratch assay to assess the effect of acute MSG stimulation on the migratory behavior of trophoblast cells and graphical representation of fold differences of the extent of area filled by cells at each time interval (**f**,**g**). All data are shown as mean ± standard deviation. Results are representative of at least three independent experiments. * *p <* 0.05; ** *p <* 0.01; ns (non-significant).

The Western blot analysis showed that MMP-2 expression was significantly downregulated on all four days, while TIMP-2 expression was upregulated on days 4, 8, and 16 (Figure 6h,j,l). MMP-9 and TIMP-1 expression were significantly upregulated on days 4 and 16 and were downregulated on Day 8. On Day 12 we observed an opposite trend for both MMP-2 and TIMP-1 expression. Moreover, MMP-9 was downregulated, and TIMP-1 was significantly upregulated on Day 12 (Figure 6i,k). Consistent with these findings, gelatin zymography revealed that the activities of MMP-2 and MMP-9 were significantly reduced in HTR-8/SVneo cells after chronic stimulation of MSG on all four days (*p* < 0.05) (Figure 7a–c). Matrigel invasion and wound healing assays performed after 16 days of chronic MSG stimulation showed a very small number of invading cells and reduced migration, respectively (Figure 7d–g). Therefore, chronic stimulation of MSG significantly downregulated the invasive and migratory properties of trophoblasts.

### 3.4. MSG Stimulation Induces OS in Cells and Tissues

**Figure 8:**
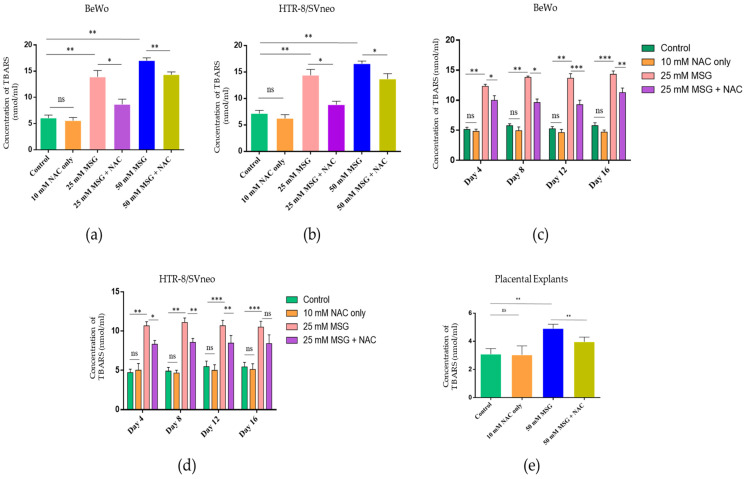
MSG induces ROS generation in trophoblast cells. TBARS was quantified using ELISA in BeWo (**a**) and HTR-8/SVneo (**b**) cells after acute stimulation of 25 and 50 mM MSG. NAC was used to quench ROS. Similarly, chronic effects of MSG stimulation in trophoblast cells was analyzed using ELISA on days 4, 8, 12 and 16, and the data are graphically represented (**c**,**d**); measurement of TBARS in the conditioned media of early placental explants treated with 50 mM MSG and 10 mM NAC (**e**). All data are presented as mean ± standard deviation. Results are representative of at least three independent experiments. * *p <* 0.05; ** *p <* 0.01; *** *p <* 0.001; ns (non-significant).

MSG elicits OS [15,40]. Therefore, we indirectly measured the concentration of TBARS generated by ROS following acute and chronic exposure of MSG in HTR-8/SVneo and BeWo cells. A significant upregulation in TBARS level was noticed in cells treated with 25 and 50 mM MSG for 24 h. To assess the role of ROS in producing TBARS, we performed rescue experiments using antioxidant NAC. Interestingly, NAC significantly downregulated TBARS production in trophoblast cells after MSG treatment (*p* < 0.01) (Figure 8a,b).

**Figure 9:**
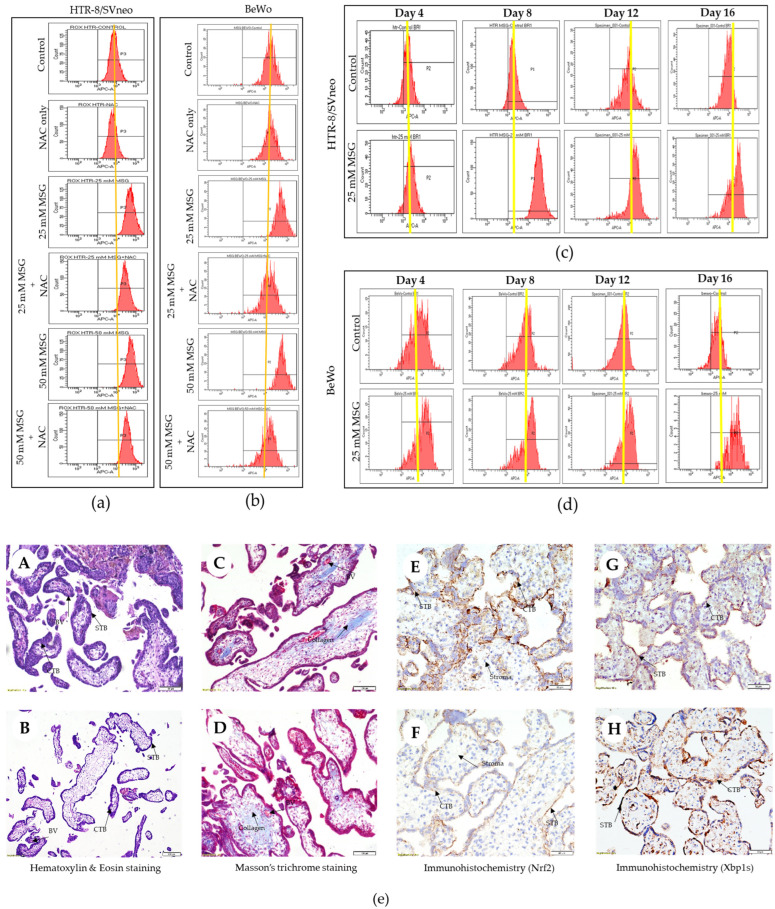
ROS production was determined in MSG-stimulated (25 and 50 mM) HTR-8/SVneo (**a**) and BeWo (**b**) trophoblast cells; n = 3. Data are represented by a shift in peak with increased ROS production. A false line was used to show the shift in peaks after MSG stimulation. ROS levels were determined in chronic 25 mM MSG-treated cells as well (**c**,**d**). All data are presented as mean ± standard deviation. The yellow line depicts the threshold limit of Control peak, peak shift towards right of it depicts ROS generation. Hematoxylin-eosin-stained sections of first-trimester placenta showing well-vascularized villi and presence of syncytial knots in the (**A**) control and (**B**) treated groups. Collagenous matrix in the stroma of the (**C**) control and (**D**) treated explants. Nrf2 immunopositivity in the (**E**) control and (**F**) treated explants. XBP1s immunopositivity in the (**G**) control and (**H**) treated explants (**e**). Results are representative of at least three independent experiments. CTB: Cytptrophoblast;, STB: Syncytiotrophoblast; BV: Blood vessel.

A similar study performed on chronic MSG treatment group revealed a significant upregulation of TBARS on all four days. In HTR-8/SVneo cells, NAC failed to quench TBARS on Day 16. In BeWo cells, a significant decrease in TBARS level was noticed on days 4, 8, 12, and 16 in the group pretreated with NAC after stimulating the cells with 25 mM MSG on every alternate day (Figure 8c,d). An increase in TBARS level was noticed in early placental explants treated with MSG, compared to that in the control group. (Figure 8e). Therefore, we stained the cells with CellRox that showed a right peak shift (increase) in cells treated with MSG in both acute and chronic conditions, as compared to that in the untreated control group. Cells pretreated with 10 mM NAC showed low levels of ROS, similar with those of the control group. The mean fluorescence intensity was measured, and cells pretreated with 10 mM NAC followed by MSG treatment showed no significant change in peak shift as compared to the group treated with MSG only, thereby suggesting that NAC could quench ROS generated in these cells (Figure 9a–d). Similarly, we confirmed the induction of OS by chronic stimulation of MSG in the early placental explants by performing an immunohistochemical analysis. In early placental explants treated with 50 mM MSG for 72 h, Nrf2 expression was significantly downregulated, and H-scores of the control and treated groups were 265 ± 16 and 120 ± 22, respectively (Figure 9e). The Western blot analysis showed significant downregulation of Nrf2 expression in both the cell lines treated with 25 mM MSG for 24 h (Figure S3a–f). Taken together, MSG treatment induced OS in trophoblast cells and early placental explants.

### 3.5. Alteration of Ultrastructural Features in Early Placental Explants and BeWo Cells by Chronic Stimulation of MSG

**Figure 10:**
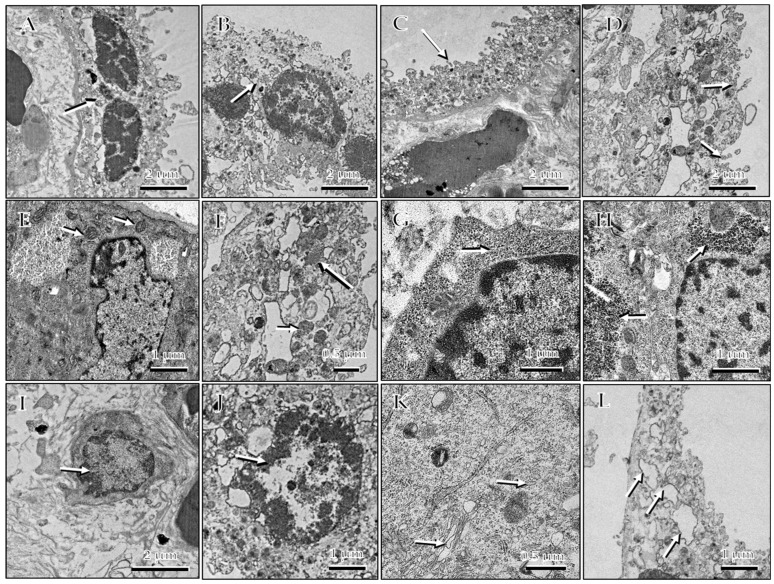
Ultrastructural changes in MSG-treated early placental explants. Transmission electron micrographs showing ultrastructural features in early placental explants. Syncytiotrophoblast in the control (**A**) and treated groups (**B**); microvilli (arrows) in the control (**C**) and treated groups (**D**); mitochondria in the control (**E**) and treated group (**F**); glycogen content (arrows) in the control (**G**) and treated groups (**H**); nucleus in the control (**I**) and treated groups (**J**); and offer in the control (**K**) and treated groups (**L**). Results are representative of at least ten independent experiments.

We speculated that ROS-mediated intracellular injury by MSG stimulation is responsible for altered trophoblast function in placental tissue explants. Using TEM, we explored the ultrastructural details of early placental tissue explants after a chronic stimulation of 50 mM MSG for 72 h. Significant ultrastructural changes were observed in placental tissue explants (n = 10). Disintegrated syncytiotrophoblast, short and distorted microvilli, disintegrated mitochondria with high glycogen content, disintegrated nuclei, and swollen endoplasmic reticulum (ER) were observed in the MSG treated group while the control group presented with completely healthy cells and intracellular organelles (Figure 10). These observations indicate that MSG caused ultrastructural changes in placental explant tissues. Moreover, we observed significant ultrastructural changes in the BeWo cells treated with 50 mM MSG for 72 h (n = 3). Disintegrated mitochondria, distorted microvilli, swollen ER, and high glycogen content were observed in MSG-treated cells (Figure S8). These results further supported our findings in treated placental explants.

### 3.6. OS Induced by MSG May Cause ER Stress in Trophoblast Cells

**Figure 11:**
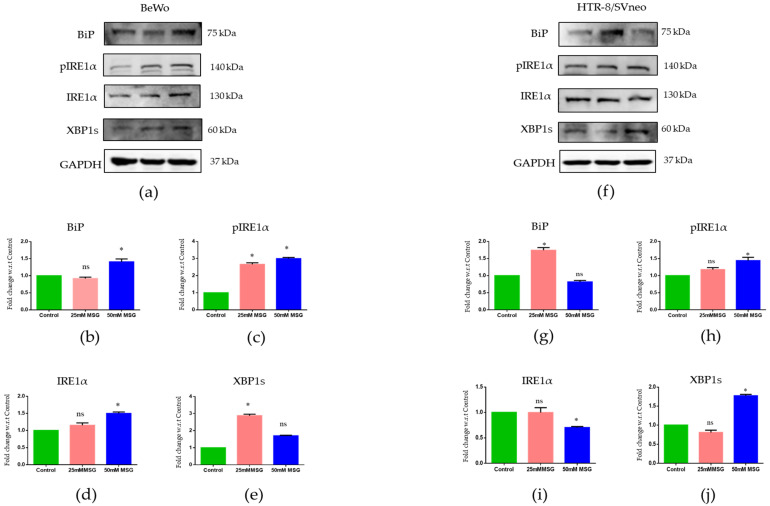
MSG induces ER stress through the UPR pathway in trophoblast cells. Western blot analysis of BiP, IRE1α, pIRE1α, and XBP-1s expression in BeWo cells (**a**); Graphical representation of band intensities quantified using ImageJ (**b**–**e**); Western blot analysis of BiP, IRE1α, and XBP-1s in HTR-8/SVneo and BeWo cells (**f**); graphical representation of band intensities quantified using ImageJ (**g**–**j**). Data are presented as mean ± standard deviation. The results shown are representative of at least three independent experiments. * *p <* 0.05; ns (non-significant).

TEM results showed that MSG treatment (50 mM) altered the ultrastructure of mitochondria and ER in the early placental explants. To investigate the role of unfolded protein response (UPR) pathway here, cells were treated with MSG for 24 h. The immunoblot analysis showed a significant upregulation of BiP in BeWo cells after 50 mM MSG treatment (Figure 11a,b). In HTR-8/SVneo cells, a significant upregulation of BiP was noticed by 25 mM MSG treatment (Figure 11f,g). In BeWo cells, phosphorylated IRE1α (pIRE1α) was significantly upregulated by 25 and 50 mM MSG treatment (Figure 11a,c); whereas, in HTR-8/SVneo cells, pIRE1α was significantly upregulated with 50 mM MSG treatment (Figure 11f,h). IRE1α expression was also significantly upregulated in BeWo cells treated with 50 mM MSG (Figure 11a,d); however, a significant downregulation was noticed in HTR-8/SVneo cells. MSG (25 mM) did not induce any significant change in IRE1α levels (Figure 11f,i). Xbp1 was significantly upregulated in BeWo cells treated with 25 mM MSG (Figure 11a,e) and in HTR-8/SVneo cells treated with 50 mM MSG (Figure 11f,j). We also checked Xbp1s expression in early placental explant tissues. Immunohistochemical analysis showed significant upregulation of Xbp1s in the MSG-treated group (H-score: 184 ± 22) compared to that in the untreated early villi explant group (H-score: 109 ± 18) (Figure 9e,g,h). These results suggest that OS caused by MSG treatment may induce ER stress in trophoblast cells.

## 4. Discussion

MSG is a majorly used food additive [41,42,43]. Although MSG is naturally present in several foods, including tomatoes, cheese, meat, and vegetables, it is mostly used as an external additive and a flavor enhancer. MSG consumption in experimental animals causes obesity, insulin resistance, reduced glucose tolerance, metabolic disorders, and disrupted energy balance [21,26,44,45,46].

This study investigated the short- and long-term effects of MSG on placental trophoblast invasion and differentiation. Using invasive extravillous trophoblasts, HTR-8/SVneo, and non-invasive villous but fusogenic BeWo cells, we explored the effects of MSG and underlying molecular mechanisms. Our study is influenced by earlier observations pointing towards a detrimental effect of MSG on the physiological system [13,33,47,48]. Although these studies reported serious health consequences of MSG, they did not explore detailed mechanistic insights. Further, the effect of MSG on the placenta and feto-maternal health has not been addressed before. The present study is an attempt in that direction.

Roman-Ramos et al. [49] reported that MSG elevates the interleukin 6 level and tumor necrosis factor-alpha-mediated inflammatory response through a microRNA-dependent pathway. Apart from its metabolic effects, MSG has a detrimental effect on the reproductive system, involving an increased number of pachytene-stage cells among the primary spermatocytes in rodents and humans [13,50]. MSG exposure in mice disrupts the basement membrane of the theca follicle in the ovary, thereby leading to atrophy and degeneration [12,13,51]. This is probably owing to enhanced OS leading to the damage of DNA and chromatin, and adduct formation in the stromal cells [17].

Despite these harmful effects of MSG on human physiology [52], no conclusive study has been undertaken to investigate the short- and long-term effects of MSG on placental function. A quick literature search in PubMed with the keywords “MSG and human placenta/trophoblast” has yielded no significant studies, thereby suggesting that not much has been investigated in this direction.

The global market for MSG is estimated at US$3.8 billion in 2020 and is projected to attain a revised size of US$4.7 billion by 2027 [53]. The MSG market in the U.S. alone is estimated at $1 billion in the year 2020. Presently, the world’s largest MSG consumer and producer is mainland China [54]. With such an enormous consumption worldwide [55] and with the possibility of its harmful effect on humans, we therefore undertook this study. Our study identified critical pathways related to trophoblast invasion and differentiation, which are perturbed upon MSG exposure in cell line-based models and placental explants. Further, MSG elicited ER stress response mediated through the BiP and IRE1α pathways in vitro. Moreover, MSG induced OS, as evidenced by elevated ROS production. This resonates with previous reports of MSG-inducing OS [31,56,57,58]. Our study also found that MSG exposure (24 h) increased the invasiveness of trophoblasts by elevating the levels of MMPs that degrade the extracellular matrix. Surprisingly, an extended MSG exposure (14 days) reduced invasion by trophoblasts. Therefore, we observed a dual effect of MSG in this aspect. This brings to us an interesting question that why MSG shows this dual response. Based on previous reports [58], we hypothesize that low levels of ROS (upon short-term MSG treatment) could enhance trophoblast invasion by multiple mechanisms including the formation of invadopodia [59] and engaging SRC family of kinases, c-Jun N-terminal kinase, and p38 kinase [60]. Moreover, ROS may activate protein kinase C by releasing intracellular calcium [61] and enhancing cell proliferation. An initial event of shallow trophoblast invasion within the decidual layer is followed by a more extended interstitial and endovascular invasion [61,62] that results in rapid maternal blood flow in the intervillous space establishing hemochorial placenta [63]. The intervillous space is oxygen-deficient before week 12 of gestation in humans. This creates transient hypoxia, which is imperative for initiating trophoblast invasion and placental angiogenesis by hypoxia-inducible factor-mediated activation of vascular endothelial growth factor [64,65]. Therefore, we expect a well-regulated step-by-step increment in oxygen level in the feto-maternal space [63,64], which should parallel with a step-by-step increase in the invasiveness of trophoblasts to maintain an adequate oxygen gradient. The importance lies in the synchrony of this entire process. A premature trophoblast invasion is as detrimental as an inadequate trophoblast invasion, with both resulting in serious consequences [66]. One way to safeguard against this premature trophoblast invasion is trophoblast-mediated sealing of uterine arteries forming plugs that prevent rapid blood flow and oxygenation at the feto-maternal interface [67].

In this study, short-term MSG treatment enhanced the migration and invasion of HTR-8/SVneo cells. We therefore speculate that MSG could potentially offset the fine balance necessary to establish physiological oxygen gradient by perturbing invasion through ROS. Long-term MSG treatment downregulated both invasion and differentiation probably owing to excessive ROS production by exhausting cellular antioxidant defense systems, as was evident by the downregulation of Nrf2, a master regulator of an antioxidant response, leading to loss of invasive potential [68] and apoptosis of these cells [69].

It is conceivable that OS could be a secondary finding in PE, but may still be a significant contributor to PE pathology (if not the only one). This statement is supported by our own lab findings as well from other studies [37,70,71,72]. That a ROS-mediated pathogenesis may contribute to PE is further justified by several clinical trials using antioxidants like melatonin [72,73] or some beneficial effect upon antioxidant administration [74]. However, we would like to state that the evidence so far linking OS to placental pathology is not yet established to be a causation and appears more of an association that is driven by other modifiers and regulators in addition to ROS.

Trophoblast invasion is a highly coordinated process involving remodeling of the maternal vasculature. The success of this process largely depends upon an orchestrated tempo-spatial effort of pro- and anti-invasive proteins [75,76]. MSG tilts this delicate balance, thereby perturbing the trophoblast invasion and differentiation program. MSG under acute exposure upregulated trophoblast differentiation markers, associated with cell–cell fusion for syncytium. In villous trophoblasts, cell–cell fusion to form syncytiotrophoblast is an important step, which is regulated by multiple drivers such as hormones, cytokines, protein kinases, and transcription factors. Short-term MSG exposure upregulated *SYN-1*, *SYN-2*, *GCM-1*, *DYSF*, *MFSD2A*, and *β-hCG*, thereby indicating abnormal syncytialization. Trophoblast differentiation is impaired in preeclamptic pregnancies as demonstrated by Fantone et al. [77].

MSG-induced ROS caused cellular damage as evident from biochemical and ultrastructural studies, and depleted Nrf2 level, as found in the placental histology. Nrf2 regulates the antioxidant defense system through multiple mechanisms [78,79,80]. Loss of Nrf2 upon MSG treatment, therefore, induces OS. We have previously observed ROS-mediated activation of UPR pathways, leading to altered trophoblast function [37]. The present study indicates that MSG probably acts through a similar mechanism. Long-term consequences of MSG consumption on placenta trophoblast cells might have an adverse effect on the conceptus though we do not have experimental evidence at this point to support our statement. Our study is preliminary in this direction. Further efforts are undertaken using rodent models to delineate in vivo effects of long-term MSG exposure on pregnancy outcomes. To confirm that the above effects are because of MSG only and not because of any osmotic changes in the cellular environment upon MSG exposure, we performed qPCR using 0.5% glycerol (Figure S9). No significant effect was observed on cell invasion and differentiation, implying an MSG-specific effect on the cells.

## 5. Conclusions

**Figure 12:**
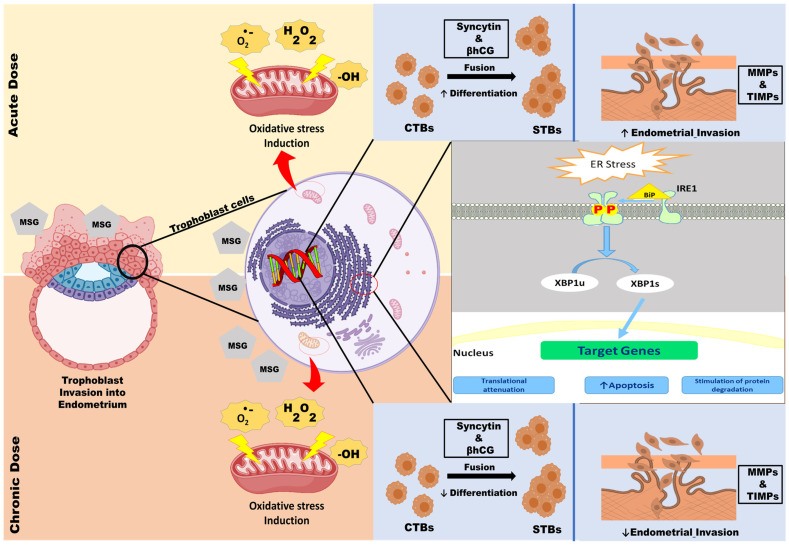
Graphical representation. The pictorial, abstract showing acute and chronic monosodium glutamate−mediated alteration in the differentiation and invasion−migration potential of trophoblasts. MSG induces oxidative stress in trophoblasts, thereby leading to endoplasmic reticulum injury and protein misfolding by inducing of unfolded protein response pathway.

MSG affected trophoblast function through ROS-mediated cellular stress. We presented evidence to show that trophoblast invasion and differentiation pathways are perturbed. Therefore, we speculate that a long-term exposure to MSG might have a detrimental effect on placentation, as represented in the graphical abstract (Figure 12). Further in vivo investigation of the effect of MSG on embryogenesis using animal models is necessary. Our study is the first report as proof of principle establishing the harmful effect of MSG on the placenta and trophoblast cells. Rodent studies are currently ongoing to explore the long-term effect of MSG through the oral route on pregnancy outcome, placental function, and possibly post-natal life. Though pregnancy in rodents does not exactly map with those of humans, the information gathered from animal studies will still be valuable in studying the effect of exposure on placentation. Our findings are novel as the effect of MSG exposure on placental trophoblasts has not yet been addressed. Identifying the possible harmful effects of MSG on trophoblasts may caution against its excessive consumption.

## Figures and Tables

**Table 1:** Antibodies used to perform Western blotting.

S.No	Name	Host	Dilution	Company	Catalog No.
1.	anti-MMP-2	Rabbit	1:1000	Cell Signaling Technology, Beverly, MA, USA	4022
2.	anti-MMP-9	Rabbit	1:1000	Cell Signaling Technology, Beverly, MA, USA	3852
3.	anti-TIMP-1	Rabbit	1:1000	Cell Signaling Technology, Beverly, MA, USA	8946
4.	anti-TIMP-2	Rabbit	1:1000	Cell Signaling Technology, Beverly, MA, USA	5738
5.	anti-Xbp1s	Rabbit	1:1000	Cell Signaling Technology, Beverly, MA, USA	40435
6.	anti-BiP	Rabbit	1:1000	ABclonal Technology, Cumming Park, Woburn, MA, USA	A0241
7.	anti-pIRE1α	Rabbit	1:1000	ABclonal Technology, Cumming Park, Woburn, MA, USA	AP0878
8.	anti-IRE1α	Rabbit	1:1000	ABclonal Technology, Cumming Park, Woburn, MA, USA	A17940
9.	anti-Nrf2	Rabbit	1:1000	Santa Cruz Biotechnology, Inc., Dallas, TX, USA	sc-365949
10.	anti-GAPDH	Rabbit	1:1500	Cell Signaling Technology, Beverly, MA, USA	5174

## Data Availability

Data is contained within the article and supplementary material.

## Supplementary Materials

The following supporting information can be downloaded at: https://www.mdpi.com/article/10.3390/antiox12030634/s1.

## Abbreviations

MSG, monosodium glutamate; PE, preeclampsia; ROS, reactive oxygen species; OS, oxidative stress; WST-1, water-soluble tetrazolium-1; SYN, syncytin;; GCM1, glial cells missing-1; DYSF, dysferlin; *β-hCG*, β subunit of human chorionic gonadotropin; SLC1A5, solute carrier family 1 member 5; MFSD2A, major facilitator superfamily domain containing 2A; MMP, matrix metalloproteinase; TIMP, tissue inhibitor of metalloproteinase; PAI-1, plasminogen activator inhibitor-1; uPA, urokinase plasminogen activator; PLAC8, placenta associated 8; RT-PCR, real time PCR; NAC, N-Acetyl cysteine; TBARS, thiobarbituric acid reactive substances; Nrf2, nuclear factor erythroid 2–related factor 2; TEM, transmission electron microscopy; ER, endoplasmic reticulum; BiP, Binding immunoglobulin protein; p-IRE1a, phosphorylated inositol-requiring enzyme 1a; IRE1a, inositol-requiring enzyme 1a; Xbp1, X-box binding protein 1; PBS, phosphate-buffered saline; PI, propidium iodide; FITC, fluorescein isothiocyanate; MDA, malondialdehyde; ECL, enhanced chemiluminescence; HRP, horseradish peroxidase; GAPDH, glyceraldehyde-3-phosphate dehydrogenase; DAPI, 4′,6-Diamidine-2′- phenylindole dihydrochloride; EDTA, Ethylenediaminetetraacetic acid; DPX, dibutylphthalate polystyrene xylene.
