# Supplementary material for: Monosodium Glutamate Perturbs Human Trophoblast Invasion and Differentiation through a Reactive Oxygen Species-Mediated Pathway: An In-Vitro Assessment
Source: Antioxidants (Basel). 2023 Mar 3;12(3):634. doi: 10.3390/antiox12030634 (PMC10045473; doi:10.3390/antiox12030634)
Supplement: Supplementary file 1 [file antioxidants-12-00634-s001.zip › Supplementary data new.pptx]

## Slide 1
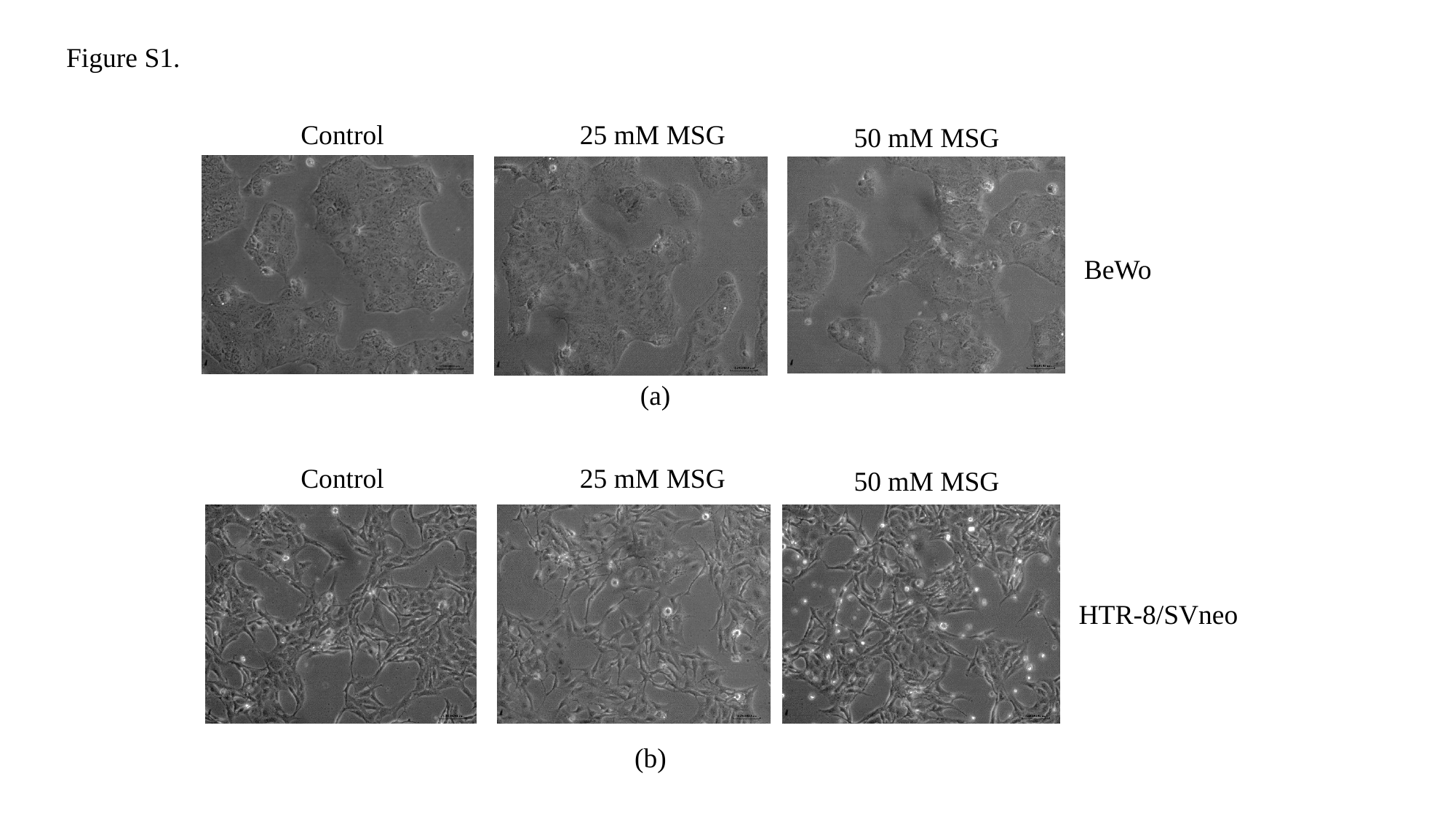

Figure S1.
Control
25 mM MSG
50 mM MSG
Control
25 mM MSG
50 mM MSG
BeWo
(a)
HTR-8/SVneo
(b)

## Slide 2
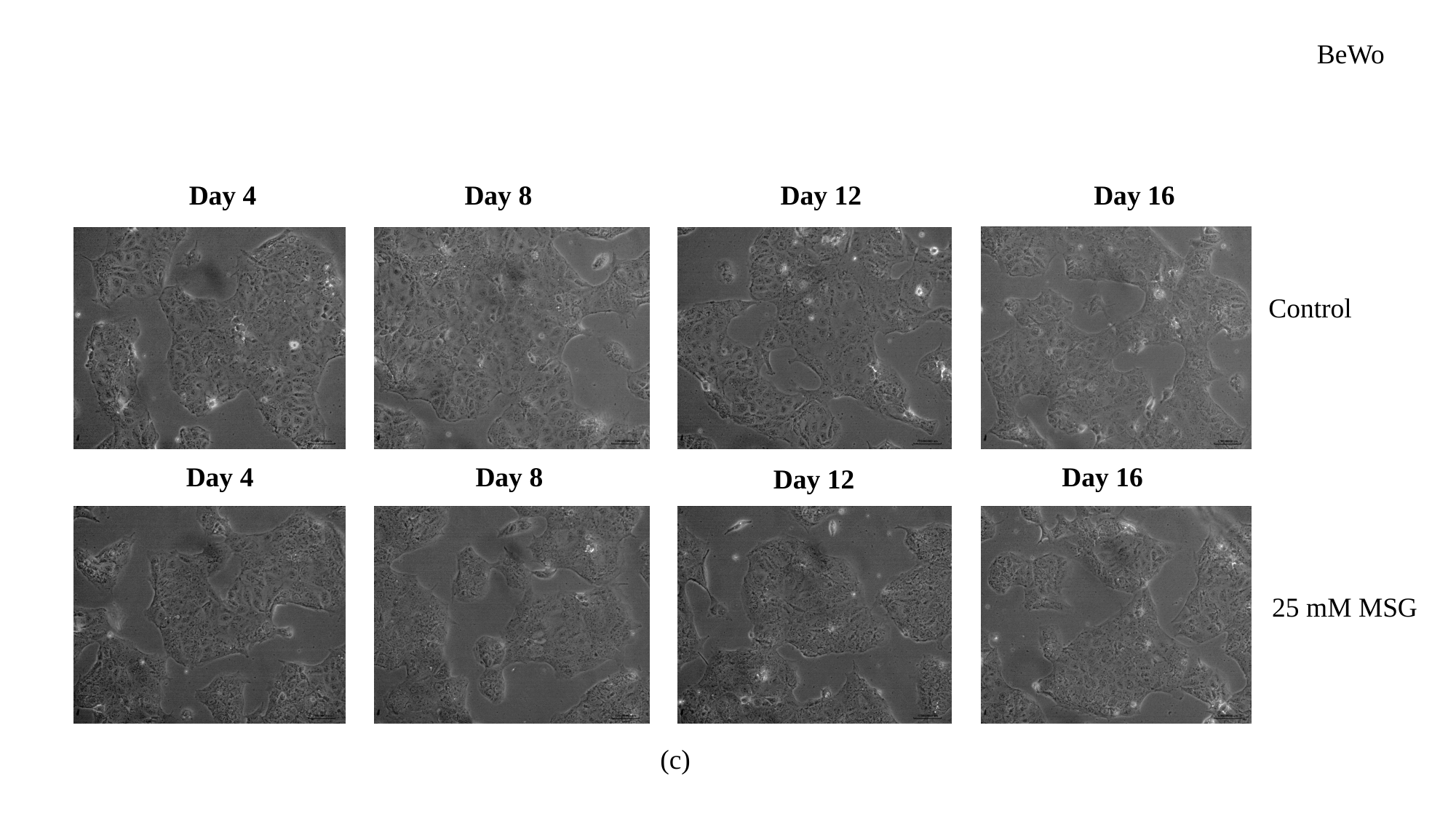

BeWo
Day 4
Day 8
Day 12
Day 16
Day 4
 Day 8
Day 16
Day 12
Control
25 mM MSG
(c)

## Slide 3
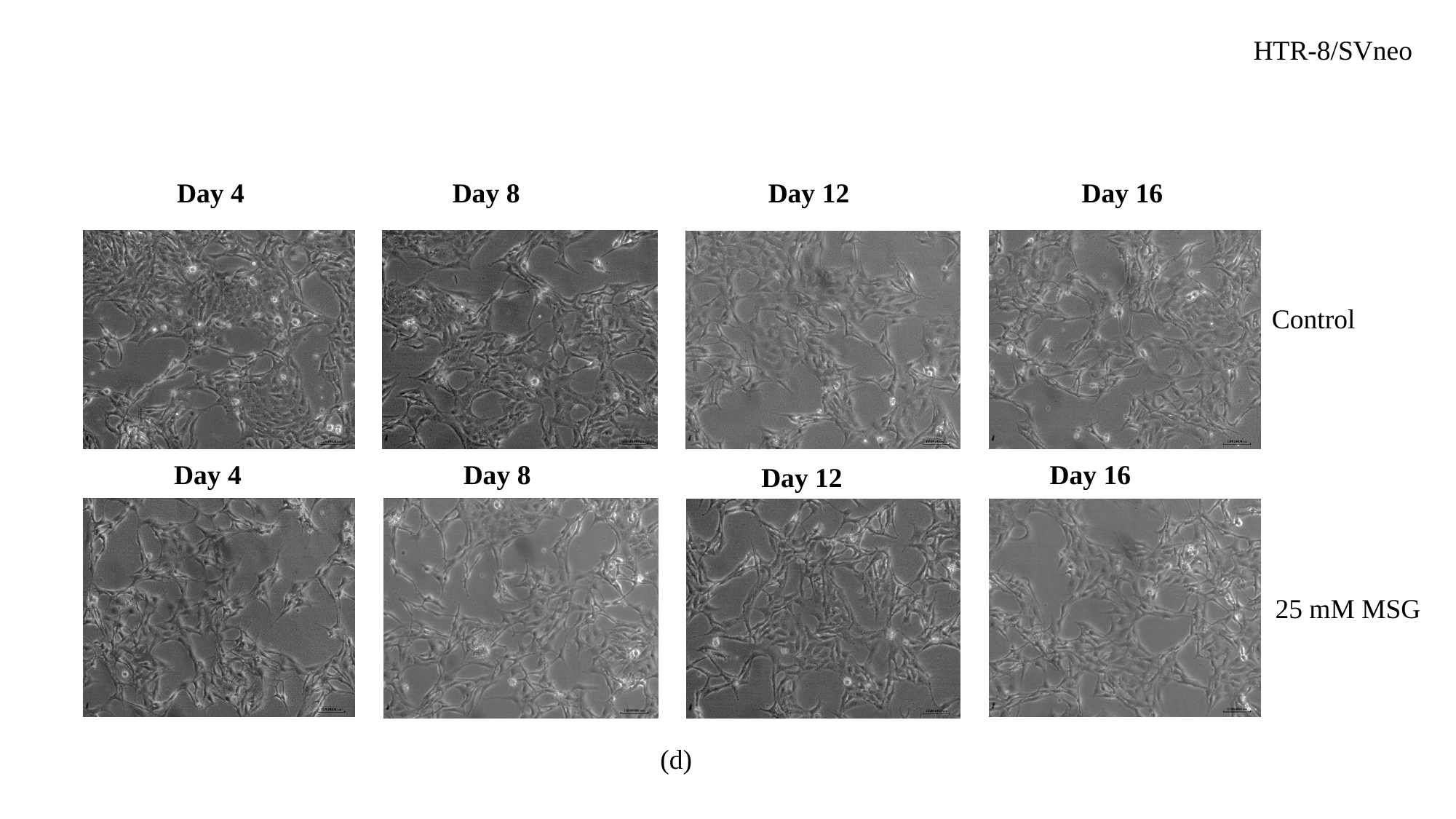

HTR-8/SVneo
Day 4
Day 8
Day 12
Day 16
Control
Day 4
 Day 8
Day 16
Day 12
25 mM MSG
(d)

## Slide 4
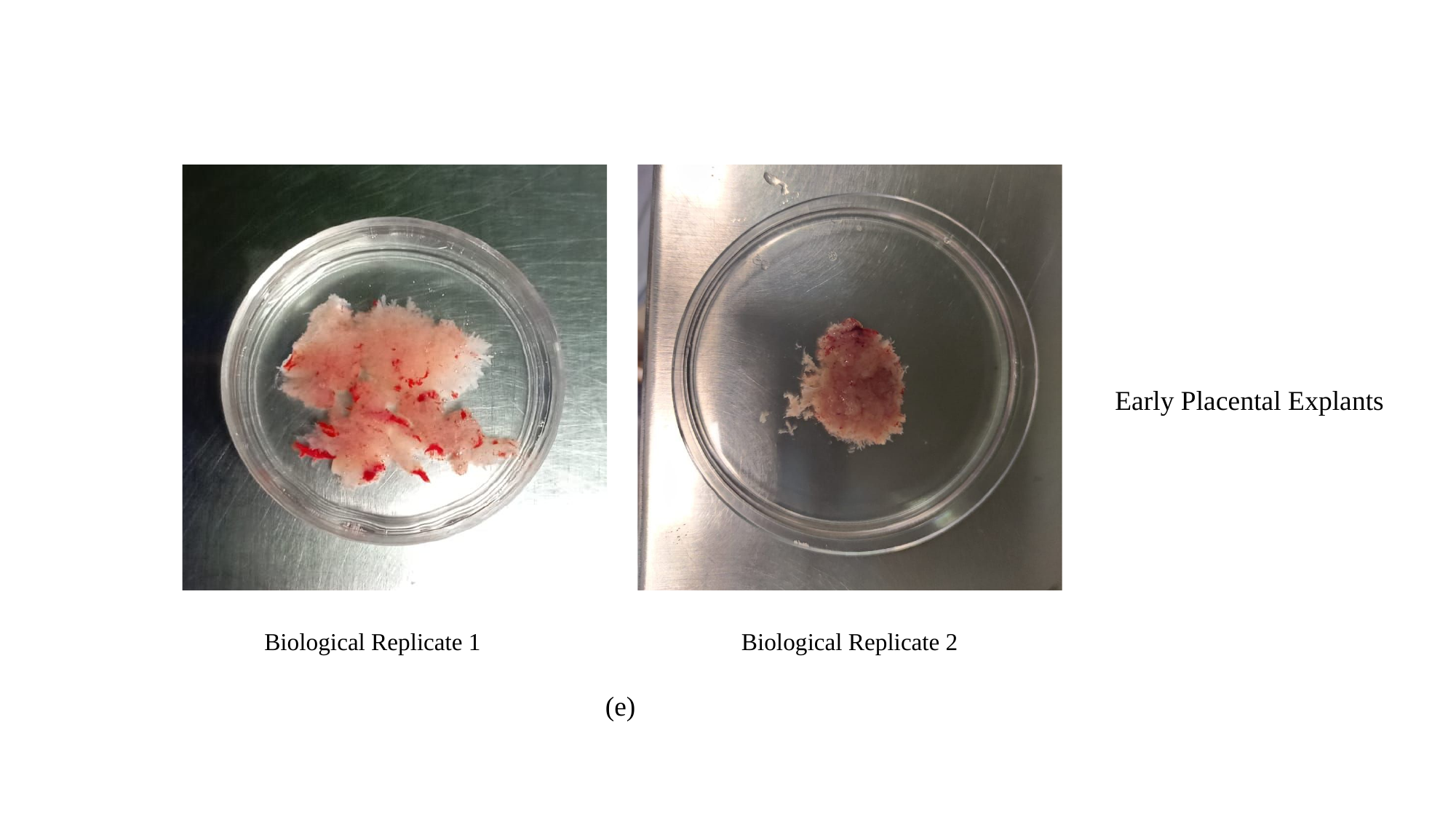

Early Placental Explants
Biological Replicate 1
Biological Replicate 2
(e)

## Slide 5
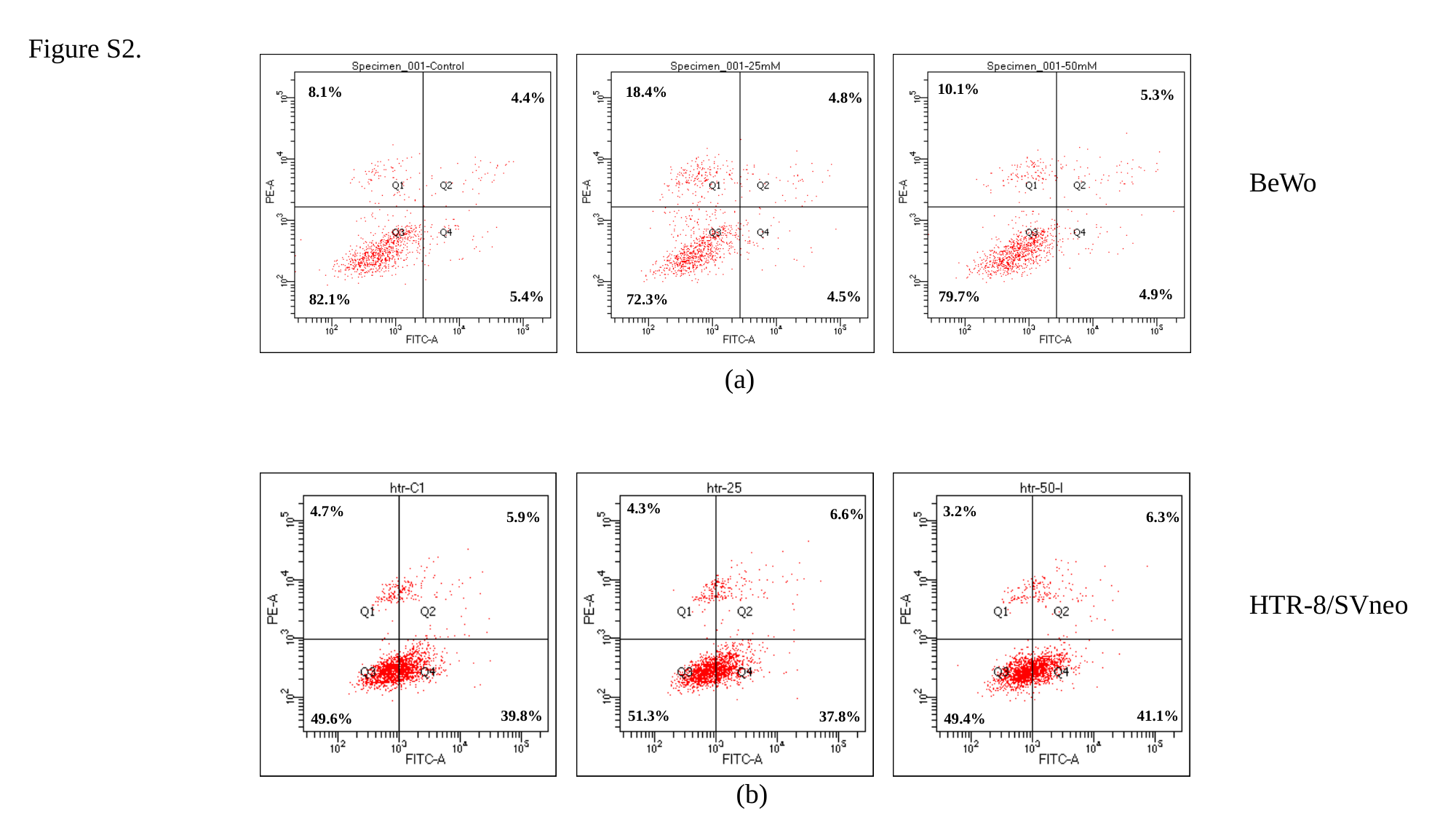

Figure S2.
10.1%
8.1%
18.4%
5.3%
4.4%
4.8%
BeWo
4.9%
5.4%
4.5%
79.7%
82.1%
72.3%
(a)
4.3%
4.7%
3.2%
6.6%
5.9%
6.3%
HTR-8/SVneo
39.8%
51.3%
41.1%
37.8%
49.6%
49.4%
(b)

## Slide 6
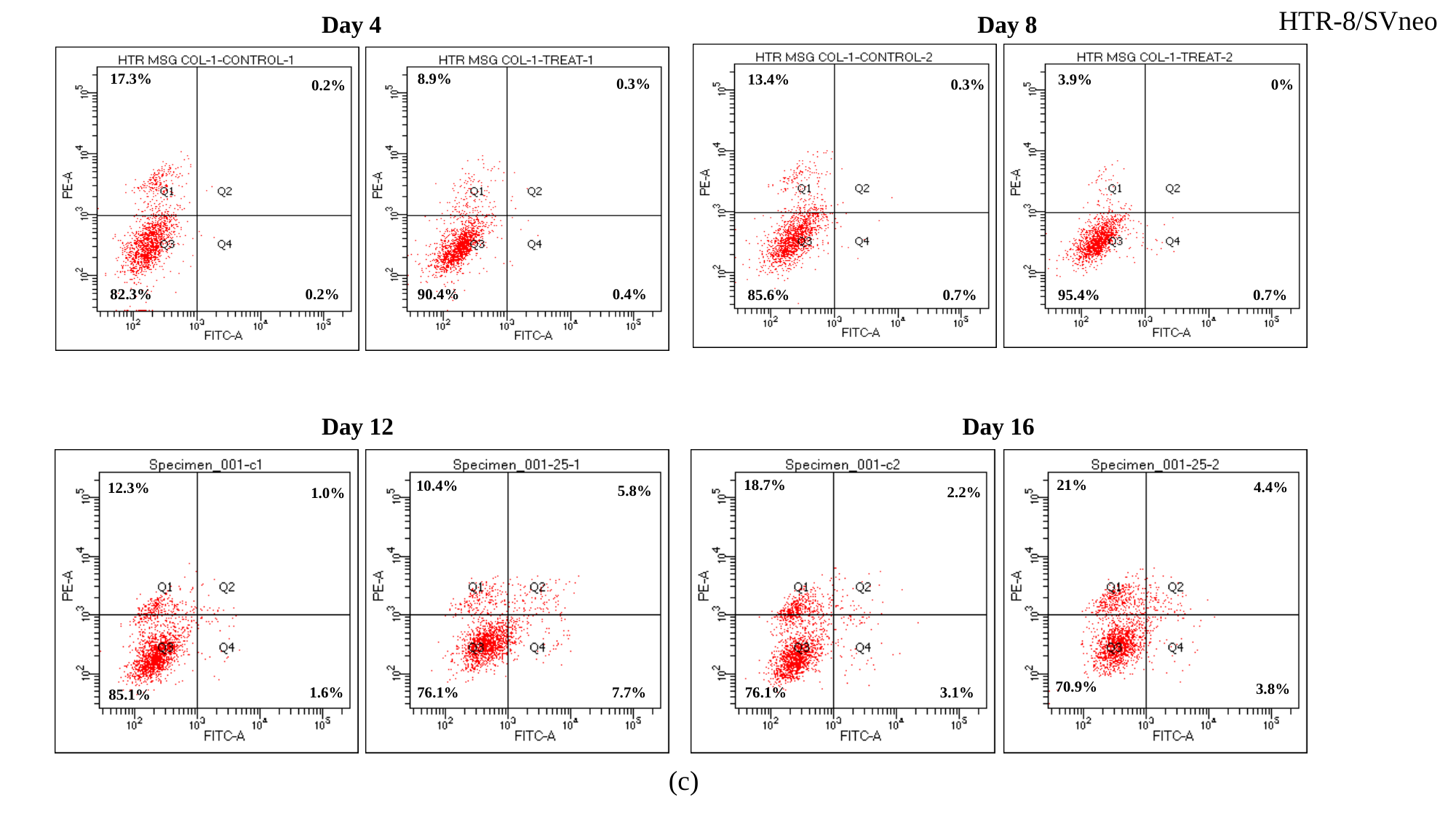

HTR-8/SVneo
Day 4
Day 8
13.4%
0.3%
0.7%
85.6%
3.9%
0%
0.7%
95.4%
8.9%
0.3%
0.4%
90.4%
17.3%
0.2%
0.2%
82.3%
Day 12
Day 16
12.3%
1.0%
1.6%
85.1%
18.7%
2.2%
3.1%
76.1%
21%
4.4%
70.9%
3.8%
10.4%
5.8%
7.7%
76.1%
(c)

## Slide 7
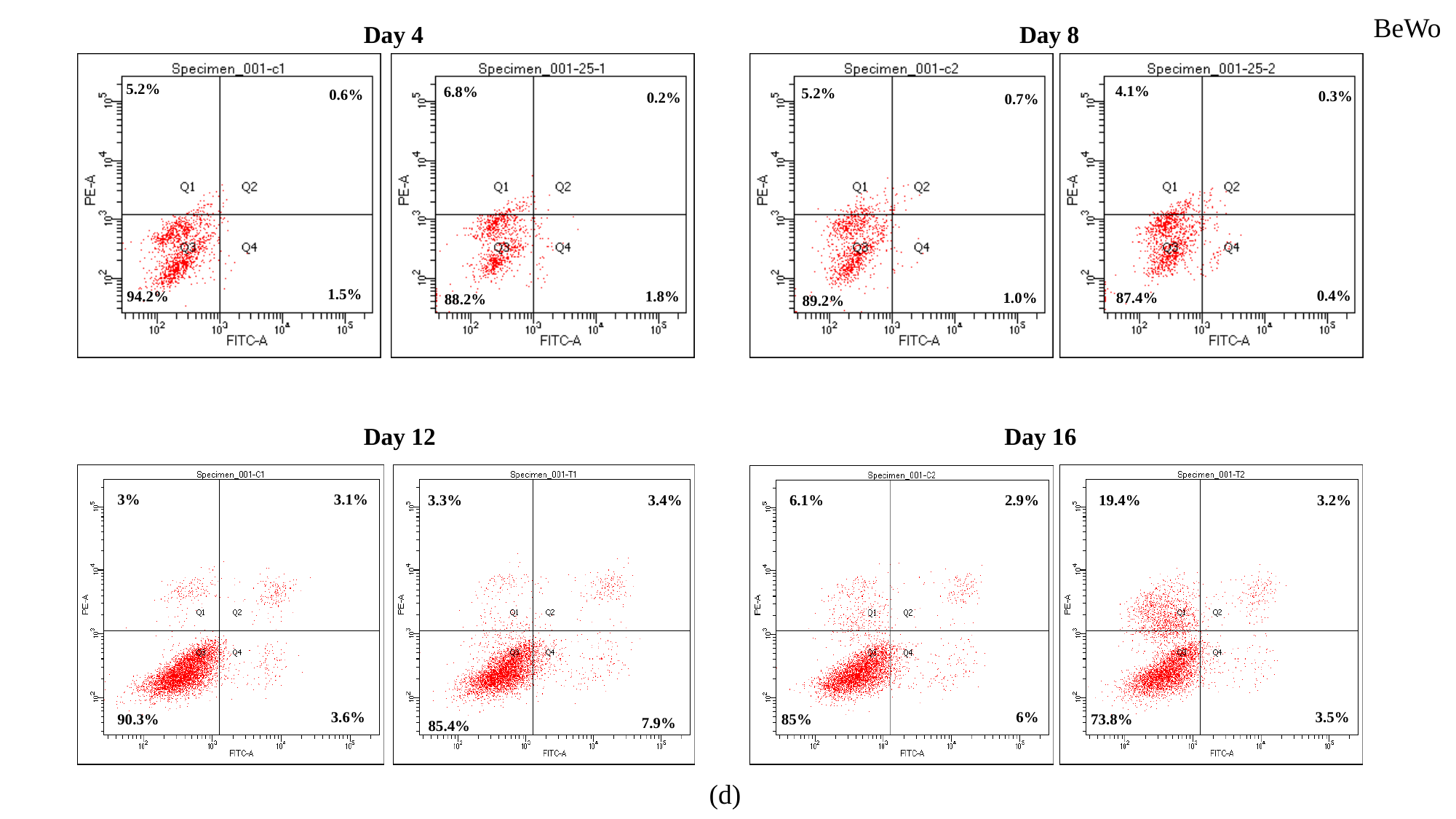

BeWo
Day 4
Day 8
4.1%
0.3%
0.4%
87.4%
6.8%
0.2%
1.8%
88.2%
5.2%
0.7%
1.0%
89.2%
5.2%
0.6%
1.5%
94.2%
Day 12
Day 16
3%
3.1%
3.3%
3.4%
6.1%
2.9%
19.4%
3.2%
6%
3.5%
3.6%
85%
73.8%
90.3%
7.9%
85.4%
(d)

## Slide 8
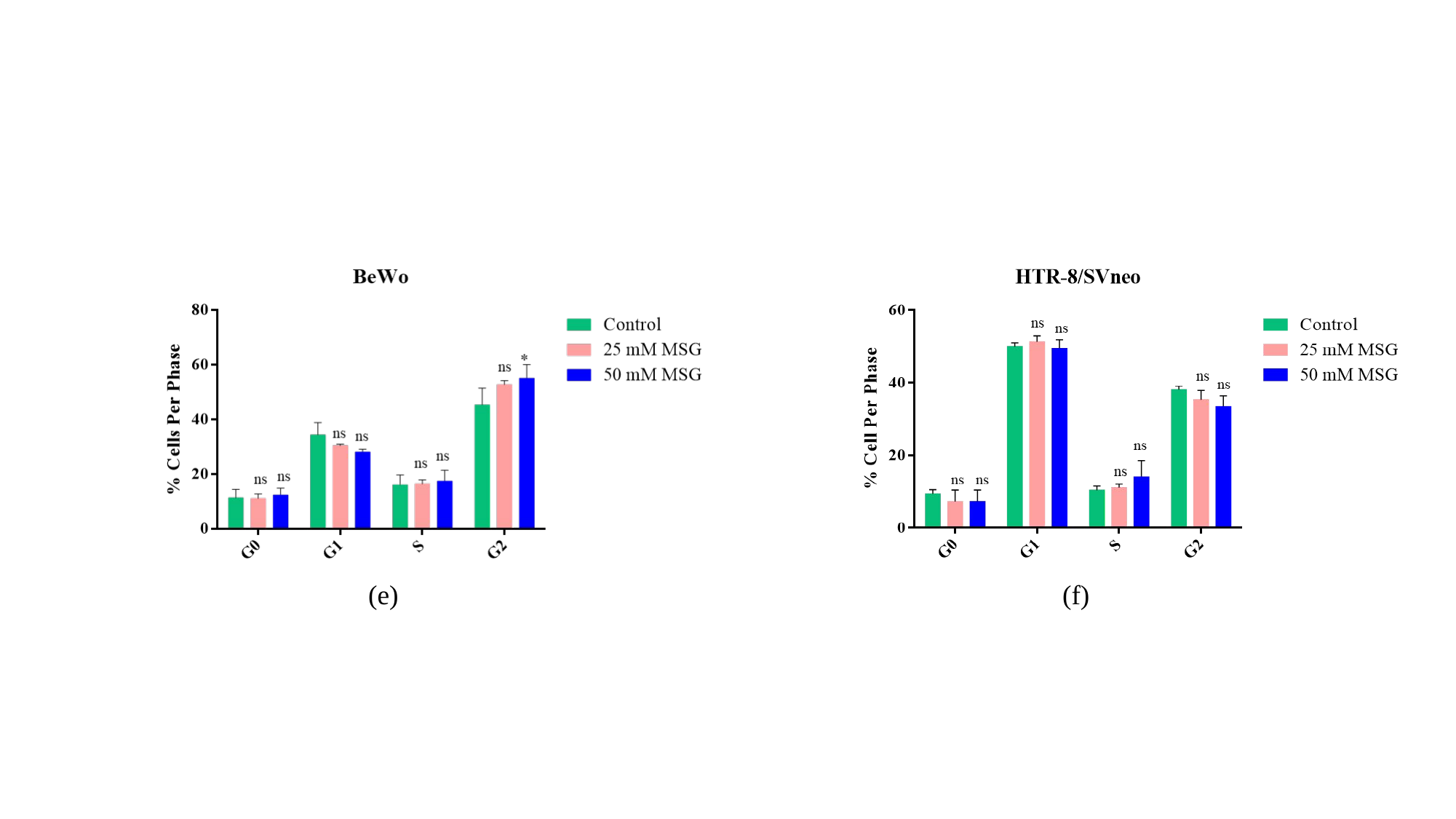

(e)
(f)

## Slide 9
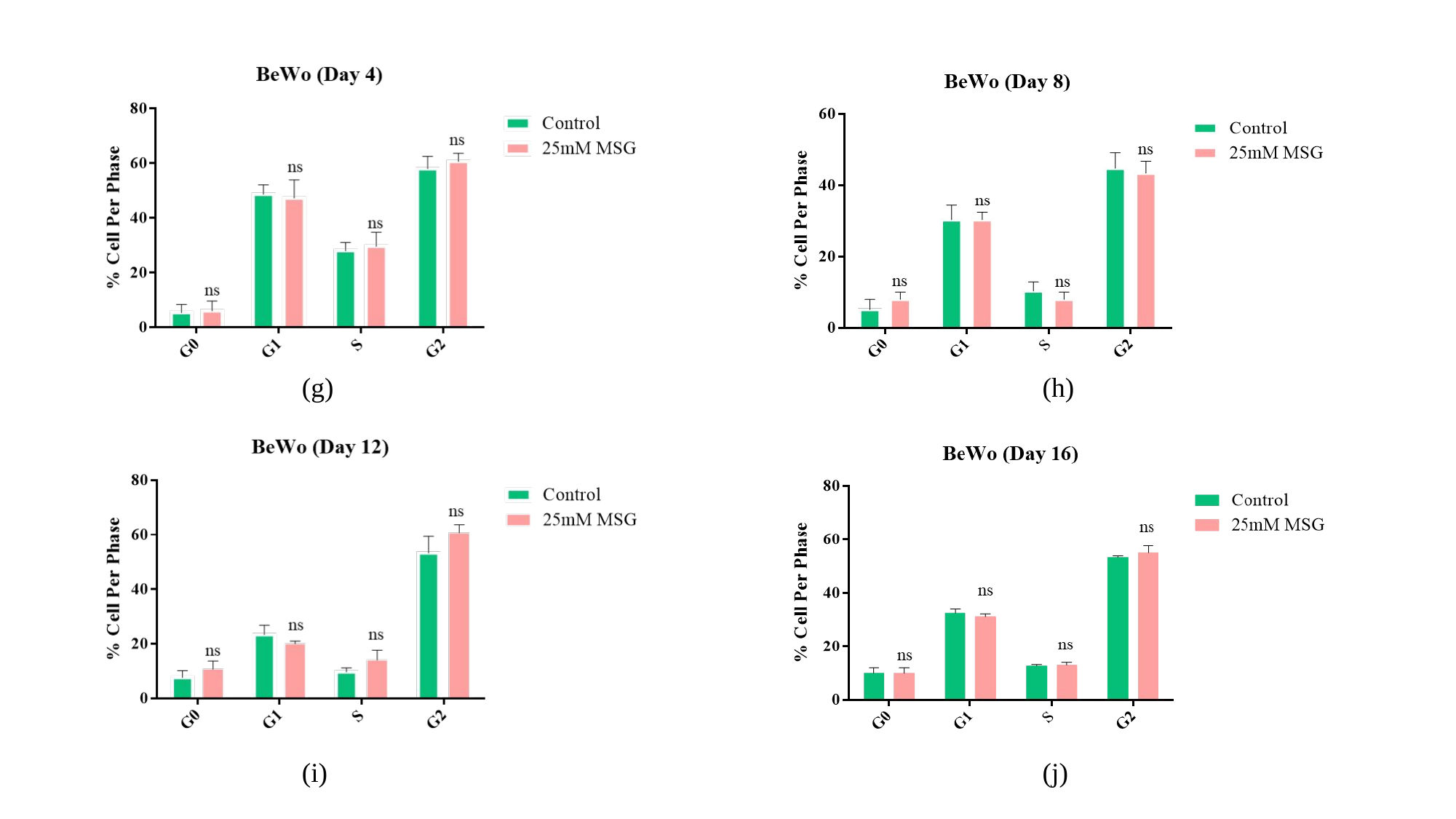

(g)
(h)
(i)
(j)

## Slide 10
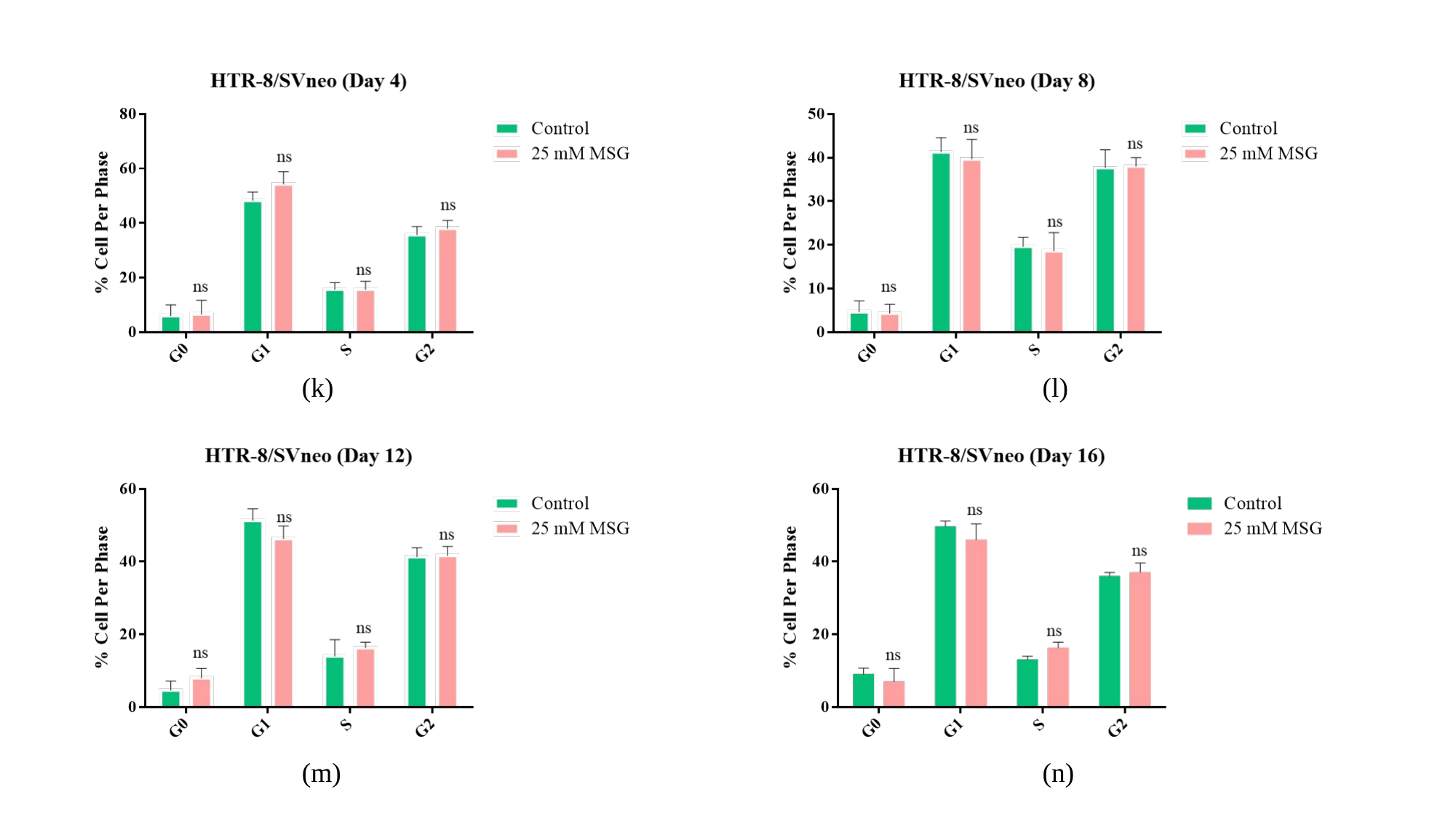

(k)
(l)
(m)
(n)

## Slide 11
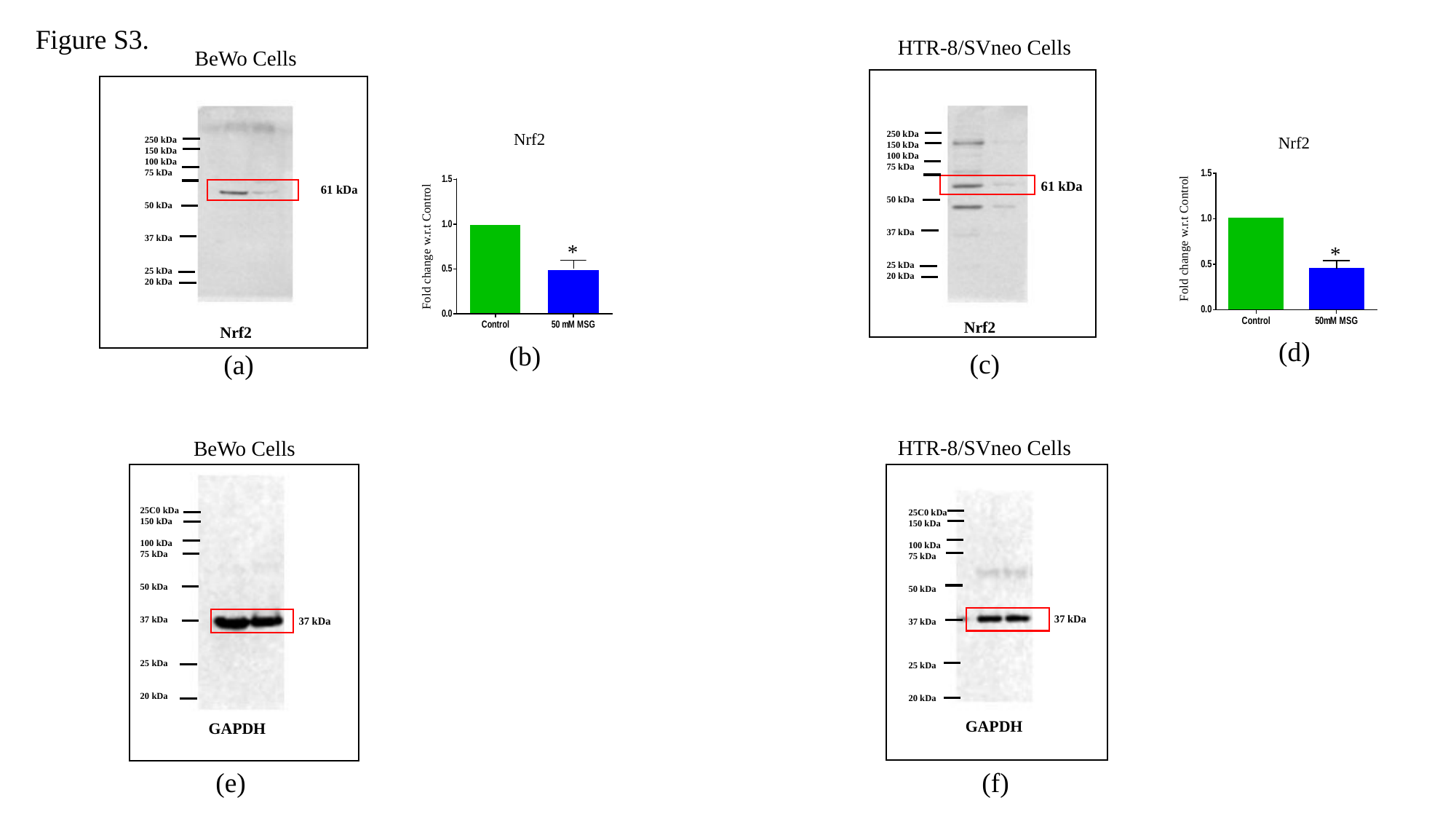

Figure S3.
HTR-8/SVneo Cells
BeWo Cells
61 kDa
Nrf2
250 kDa
150 kDa
100 kDa
75 kDa
50 kDa
37 kDa
25 kDa
20 kDa
Nrf2
Nrf2
250 kDa
150 kDa
100 kDa
75 kDa
50 kDa
37 kDa
25 kDa
20 kDa
*
(d)
*
(b)
61 kDa
Fold change w.r.t Control
Fold change w.r.t Control
Nrf2
(c)
(a)
HTR-8/SVneo Cells
BeWo Cells
25C0 kDa
150 kDa
100 kDa
75 kDa
50 kDa
37 kDa
25 kDa
20 kDa
25C0 kDa
150 kDa
100 kDa
75 kDa
50 kDa
37 kDa
25 kDa
20 kDa
37 kDa
37 kDa
GAPDH
GAPDH
(e)
(f)

## Slide 12
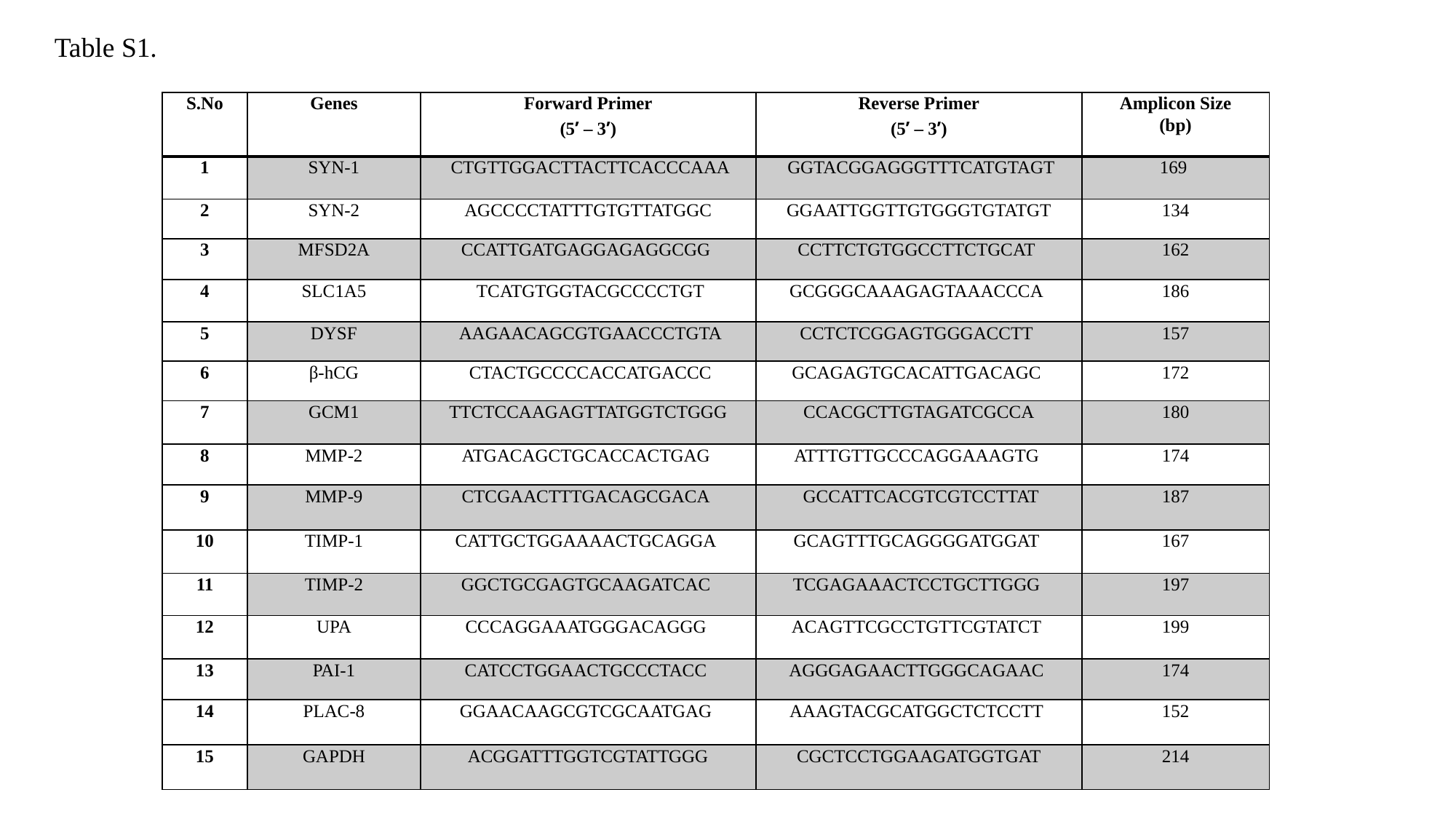

Table S1.
| S.No | Genes | Forward Primer (5’ – 3’) | Reverse Primer (5’ – 3’) | Amplicon Size (bp) |
| --- | --- | --- | --- | --- |
| 1 | SYN-1 | CTGTTGGACTTACTTCACCCAAA | GGTACGGAGGGTTTCATGTAGT | 169 |
| 2 | SYN-2 | AGCCCCTATTTGTGTTATGGC | GGAATTGGTTGTGGGTGTATGT | 134 |
| 3 | MFSD2A | CCATTGATGAGGAGAGGCGG | CCTTCTGTGGCCTTCTGCAT | 162 |
| 4 | SLC1A5 | TCATGTGGTACGCCCCTGT | GCGGGCAAAGAGTAAACCCA | 186 |
| 5 | DYSF | AAGAACAGCGTGAACCCTGTA | CCTCTCGGAGTGGGACCTT | 157 |
| 6 | β-hCG | CTACTGCCCCACCATGACCC | GCAGAGTGCACATTGACAGC | 172 |
| 7 | GCM1 | TTCTCCAAGAGTTATGGTCTGGG | CCACGCTTGTAGATCGCCA | 180 |
| 8 | MMP-2 | ATGACAGCTGCACCACTGAG | ATTTGTTGCCCAGGAAAGTG | 174 |
| 9 | MMP-9 | CTCGAACTTTGACAGCGACA | GCCATTCACGTCGTCCTTAT | 187 |
| 10 | TIMP-1 | CATTGCTGGAAAACTGCAGGA | GCAGTTTGCAGGGGATGGAT | 167 |
| 11 | TIMP-2 | GGCTGCGAGTGCAAGATCAC | TCGAGAAACTCCTGCTTGGG | 197 |
| 12 | UPA | CCCAGGAAATGGGACAGGG | ACAGTTCGCCTGTTCGTATCT | 199 |
| 13 | PAI-1 | CATCCTGGAACTGCCCTACC | AGGGAGAACTTGGGCAGAAC | 174 |
| 14 | PLAC-8 | GGAACAAGCGTCGCAATGAG | AAAGTACGCATGGCTCTCCTT | 152 |
| 15 | GAPDH | ACGGATTTGGTCGTATTGGG | CGCTCCTGGAAGATGGTGAT | 214 |

## Slide 13
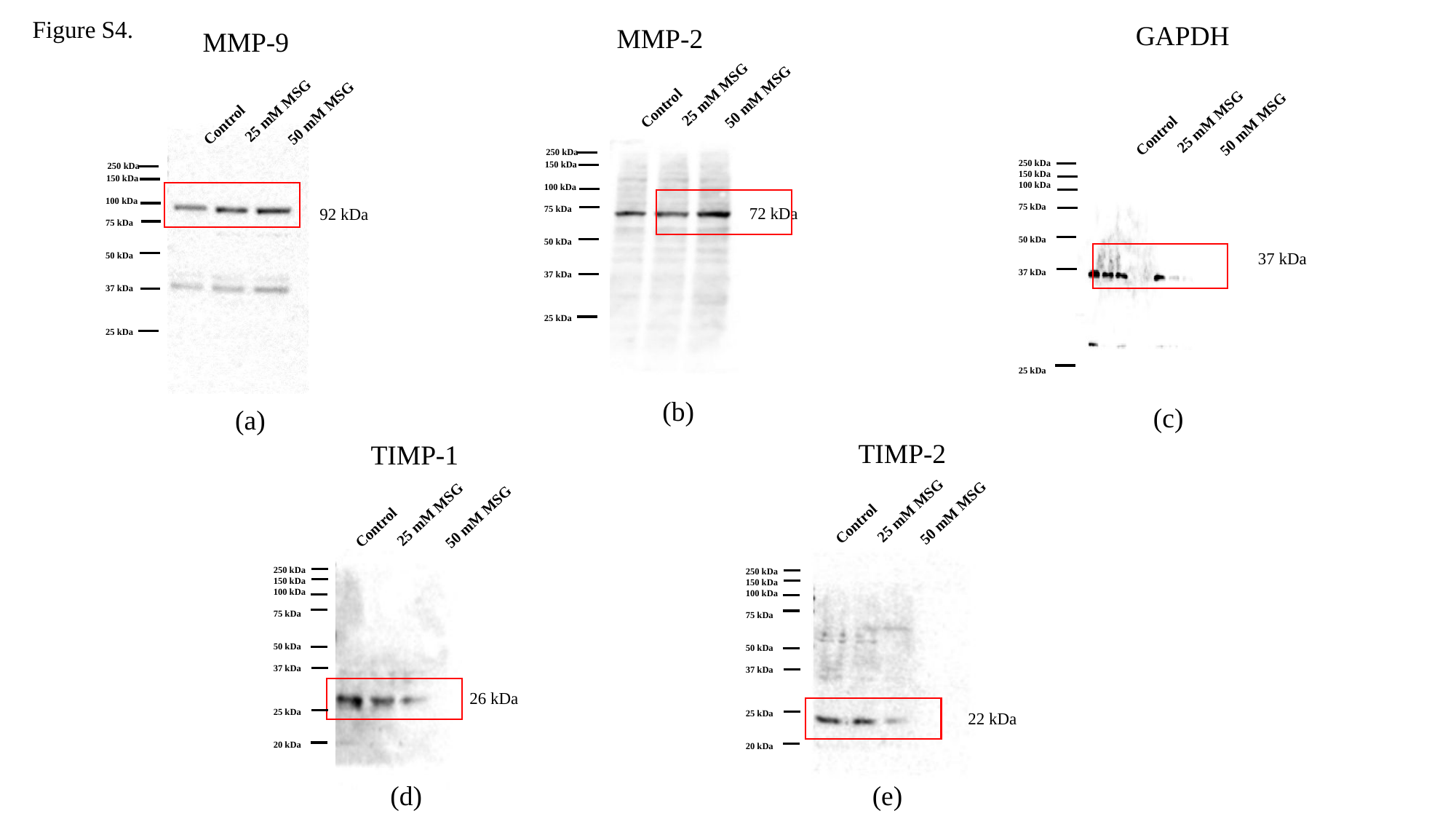

Figure S4.
GAPDH
37 kDa
25 mM MSG
50 mM MSG
Control
250 kDa
150 kDa
100 kDa
75 kDa
50 kDa
37 kDa
25 kDa
MMP-2
72 kDa
25 mM MSG
50 mM MSG
Control
250 kDa
100 kDa
75 kDa
50 kDa
37 kDa
25 kDa
150 kDa
MMP-9
92 kDa
25 mM MSG
50 mM MSG
Control
250 kDa
100 kDa
75 kDa
50 kDa
37 kDa
25 kDa
150 kDa
(b)
(c)
(a)
TIMP-2
22 kDa
25 mM MSG
50 mM MSG
Control
250 kDa
150 kDa
100 kDa
75 kDa
50 kDa
37 kDa
25 kDa
20 kDa
TIMP-1
26 kDa
25 mM MSG
50 mM MSG
Control
250 kDa
150 kDa
100 kDa
75 kDa
50 kDa
37 kDa
25 kDa
20 kDa
(d)
(e)

## Slide 14
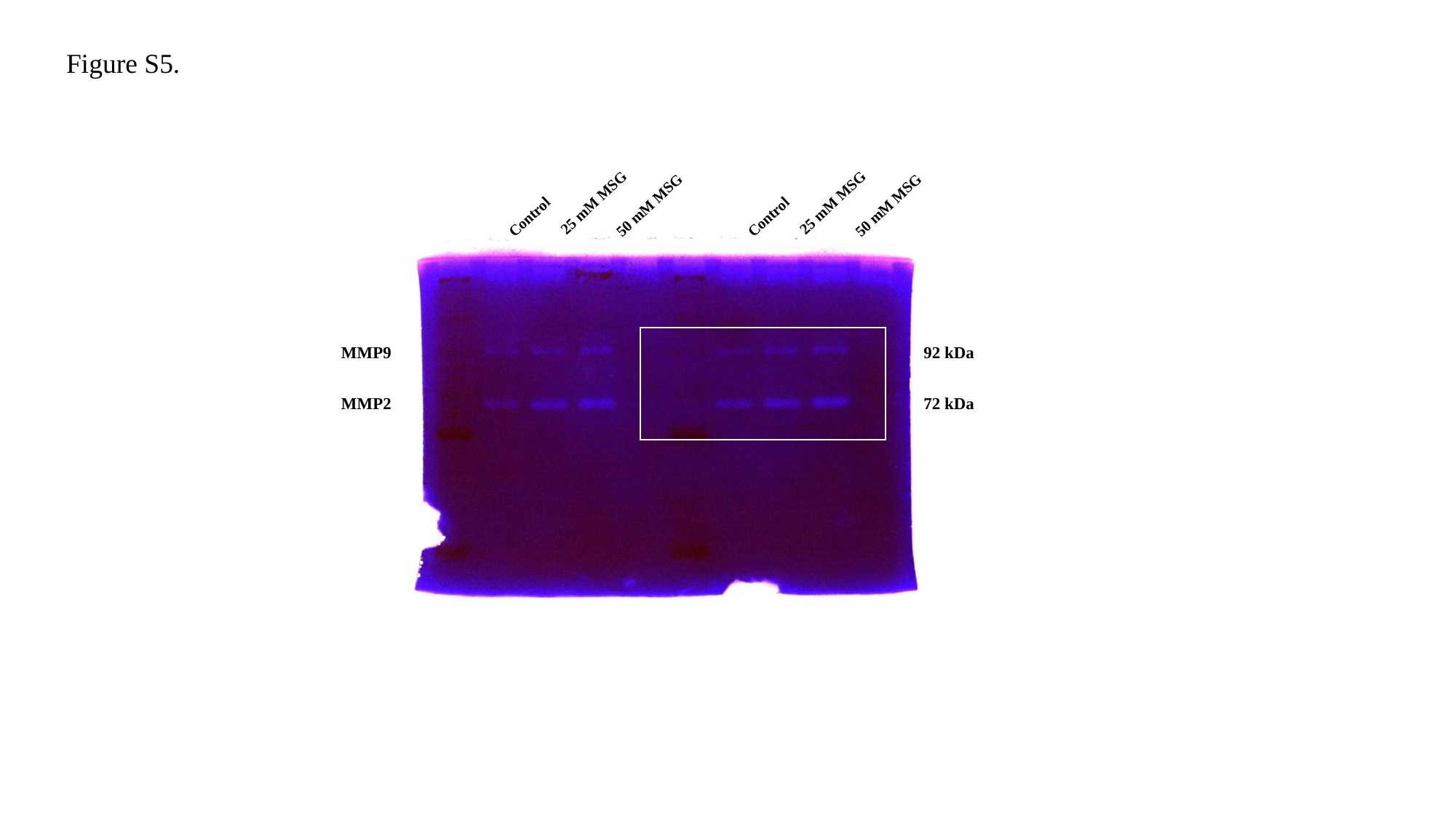

Figure S5.
25 mM MSG
25 mM MSG
50 mM MSG
50 mM MSG
Control
Control
MMP9
92 kDa
MMP2
72 kDa

## Slide 15
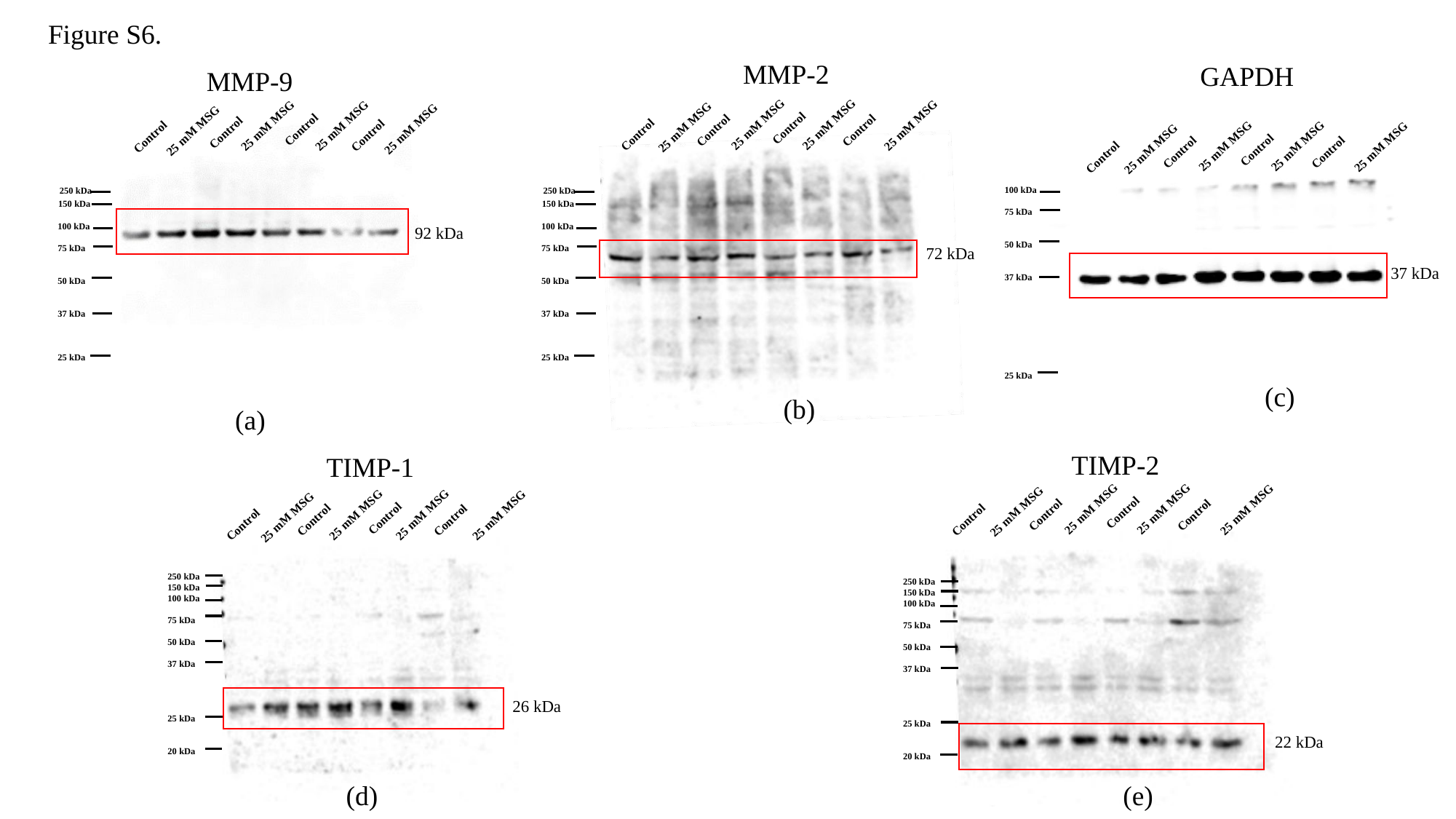

Figure S6.
MMP-2
25 mM MSG
25 mM MSG
25 mM MSG
25 mM MSG
Control
Control
Control
Control
72 kDa
250 kDa
100 kDa
75 kDa
50 kDa
37 kDa
25 kDa
150 kDa
GAPDH
37 kDa
25 mM MSG
25 mM MSG
25 mM MSG
25 mM MSG
Control
Control
Control
Control
100 kDa
75 kDa
50 kDa
37 kDa
25 kDa
MMP-9
92 kDa
25 mM MSG
25 mM MSG
25 mM MSG
Control
25 mM MSG
Control
Control
Control
250 kDa
100 kDa
75 kDa
50 kDa
37 kDa
25 kDa
150 kDa
(c)
(b)
(a)
TIMP-2
22 kDa
25 mM MSG
25 mM MSG
25 mM MSG
25 mM MSG
Control
Control
Control
Control
TIMP-1
26 kDa
25 mM MSG
25 mM MSG
25 mM MSG
25 mM MSG
Control
Control
Control
Control
250 kDa
150 kDa
100 kDa
75 kDa
50 kDa
37 kDa
25 kDa
20 kDa
250 kDa
150 kDa
100 kDa
75 kDa
50 kDa
37 kDa
25 kDa
20 kDa
(d)
(e)

## Slide 16
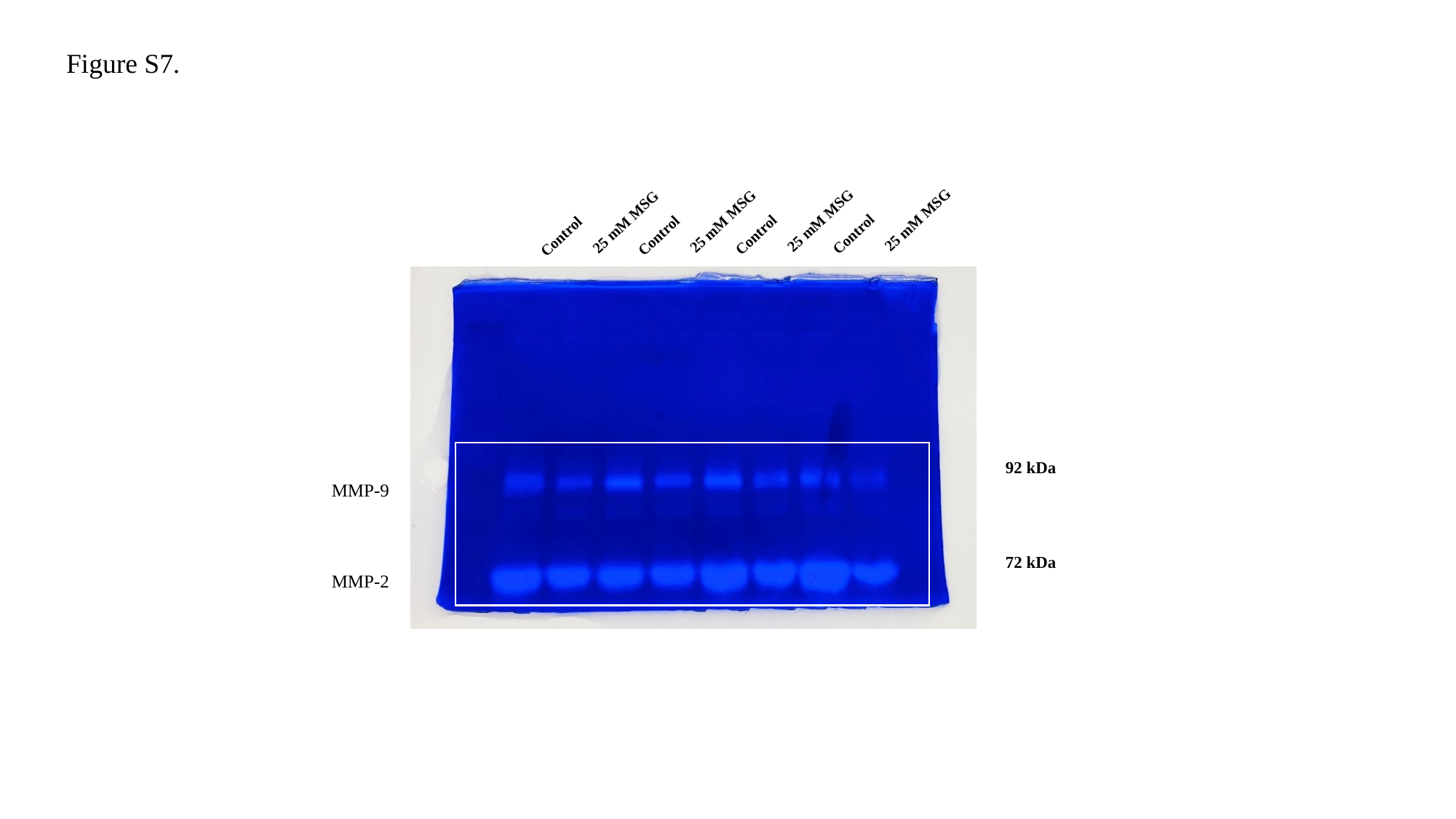

Figure S7.
25 mM MSG
25 mM MSG
25 mM MSG
25 mM MSG
Control
Control
Control
Control
92 kDa
MMP-9
72 kDa
MMP-2

## Slide 17
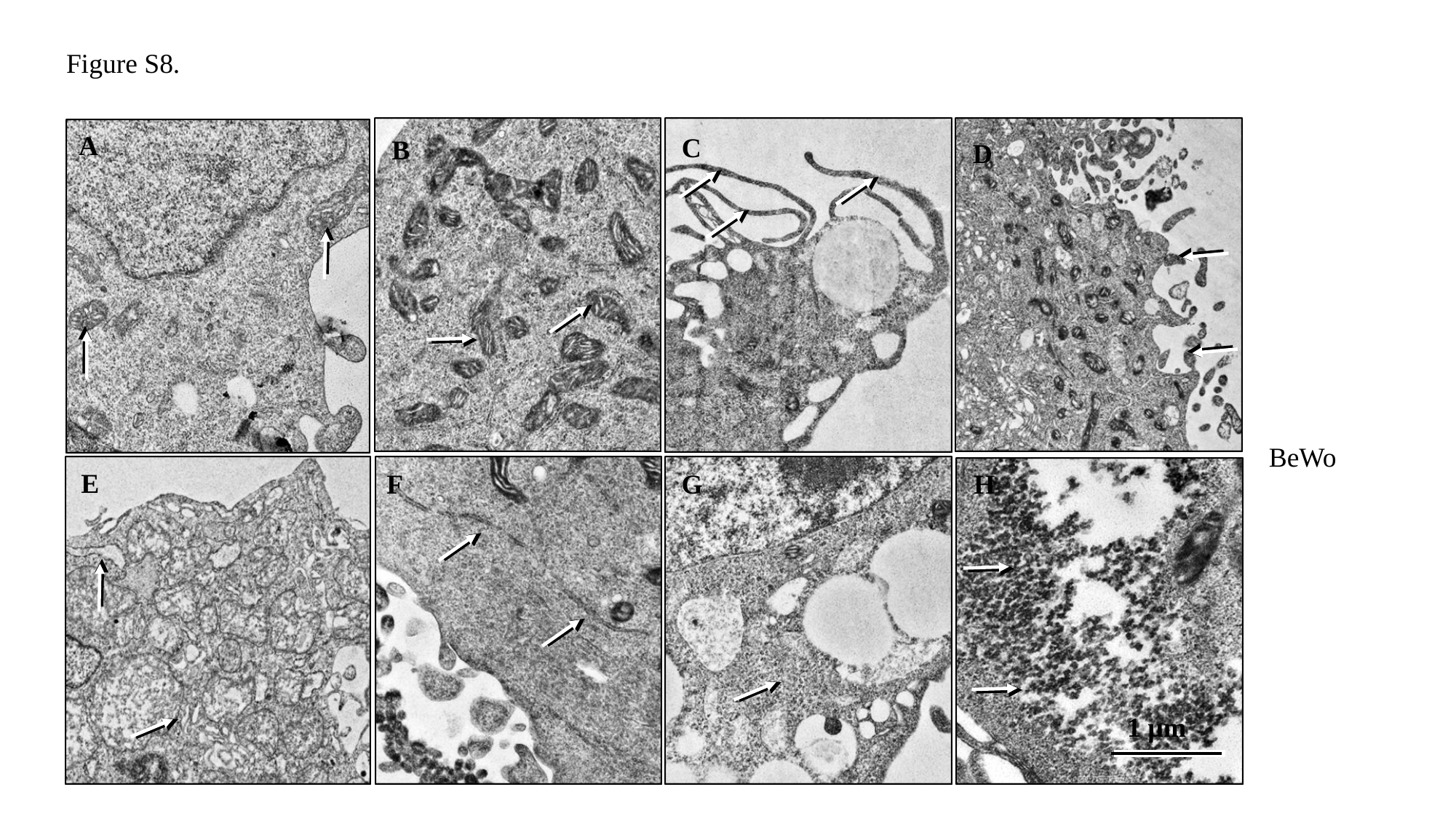

Figure S8.
A
C
B
D
E
H
F
G
1 µm
BeWo

## Slide 18
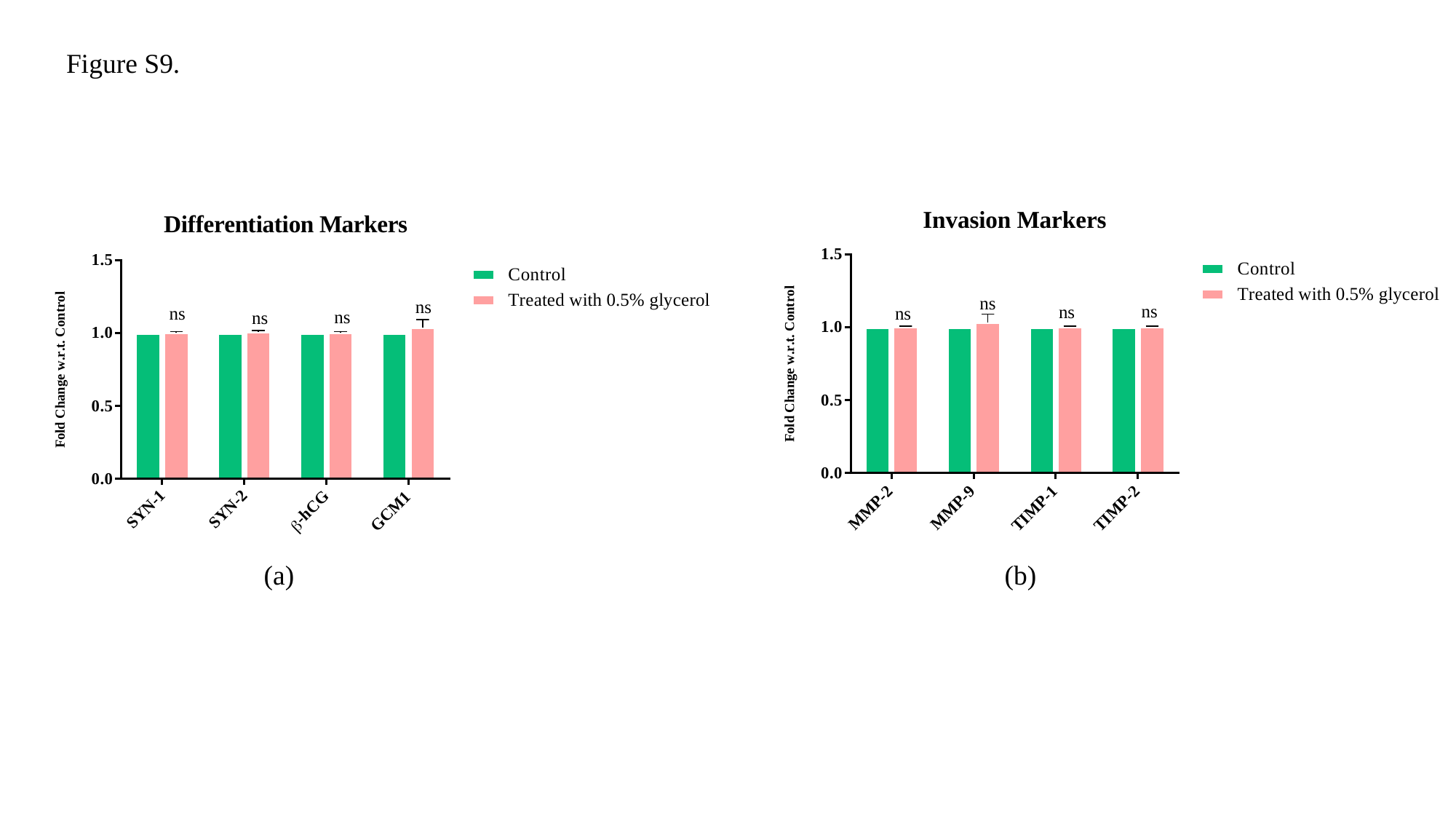

Figure S9.
ns
ns
ns
ns
ns
ns
ns
ns
(a)
(b)
