# Supplementary material for: Monosodium Glutamate Perturbs Human Trophoblast Invasion and Differentiation through a Reactive Oxygen Species-Mediated Pathway: An In-Vitro Assessment
Source: Antioxidants (Basel). 2023 Mar 3;12(3):634. doi: 10.3390/antiox12030634 (PMC10045473; doi:10.3390/antiox12030634)
Supplement: Supplementary file 1 [file antioxidants-12-00634-s001.zip › Supplementary material.pdf]

SUPPLEMENTARY DETAILS

Legends

Figure S1. (a) BeWo and (b) HTR-8/SVneo cells were treated with 25 mM MSG and 50 mM MSG to check their acute effects on the trophoblast cells. The images were visualized under an inverted microscope at 100X magnification. The trophoblast cells were treated with 25 mM MSG alternatively for a stretch of 16 days and images of (c) BeWo and (d) HTR-8/SVneo cells were visualized under the microscope on days 4, 8, 12 and 16 at 100X magnification to check the effect of chronic stimulation of MSG. (e) Images of treated and untreated early placental explants stimulated with 50 mM MSG.

Figure S2. (a-d) Cell death was measured by flow cytometry using Annexin V-FITC and PI double staining. BeWo and HTR-8/SVneo cells were exposed to the chosen concentrations of MSG. In each density plot quadrant Q1: shows necrotic cells (Annexin− PI+); Q2: late apoptotic cells (Annexin+ PI+); Q3: shows the viable cells (Annexin− PI−) and Q4: early apoptotic cells (Annexin+ PI−) (e-f) Cell cycle analysis was performed in the trophoblast cells using the chosen concentration of MSG for acute stimulation of 24h to check the percentage of cell proliferation in each phase of cell cycle. (g-n) Similarly, cell cycle analysis was performed in the trophoblast cells using the chosen concentration of MSG for chronic stimulation of 16 days to check the percentage of cell proliferation in each phase of cell cycle on Day4, Day 8, Day 12 and Day 16. Results are representative of at least three independent experiments. * $p < 0.05$; ** $p < 0.01$; *** $p < 0.001$. **** $p < 0.0001$

Figure S3. (a-f) Protein expression of Nrf2 was also checked in the lysates of the trophoblast cells BeWo and HTR-8/SVneo treated with 50 mM MSG to check the chronic effect of MSG on Nrf2. The data was normalized with respect to GAPDH. Band intensities were quantified using ImageJ and plotted graphically. All data obtained are shown as Mean ± Standard deviation. The results shown are representative of at least three independent experiments. * $p < 0.05$; ** $p < 0.01$; *** $p < 0.001$. **** $p < 0.0001$

Figure S4. (a-e) Original obtained after performing western blotting showing the protein levels of MMPs and their inhibitors after acute stimulation of MSG in HTR-8/SVneo cells. GAPDH served as the loading control.

Figure S5. Original gel obtained after performing gelatin zymography showing the levels of MMPs and their TIMPs after acute stimulation of MSG in the conditioned media of HTR-8/SVneo cells.

Figure S6. (a-e) Original blots obtained after performing western blotting the protein levels of MMPs and their inhibitors after chronic stimulation of MSG in HTR-8/SVneo cells. GAPDH served as the loading control.

Figure S7. Original gel obtained after performing gelatin zymography showing the levels of MMPs and their TIMPs after chronic stimulation of MSG in the conditioned media of HTR-8/SVneo cells.

Figure S8. Ultrastructural changes in 50 mM MSG treated BeWo compared to the untreated control group incubated for 72h. Transmission electron micrographs showing ultrastructural features in the early placental explant samples. **(a,b)** Showing mitochondria in control (A) and treated group (B), in the latter, dark, condensed mitochondria are seen (arrows). **(c, d)** Microvilli in control (C) and treated groups (D); in the latter, they were tiny, reduced, and fragmented (arrows). **(e, f)** The MSG-treated tissue explants had distorted, and swollen RER (F), compared to those in control (E, arrows). **(g, h)** Glycogen content (arrows) was high in the MSG-treated tissues (H), compared to that in control (G). Results are representative of at least three independent experiments. Scale bar (shown in H) applies to all other figures.

Figure S9. (a) BeWo and (b) HTR-8/SVneo cells were treated with 0.5% glycerol to rule out the possibility that the effects on trophoblast invasion and differentiation are due to MSG only and not due to any osmotic changes in the cellular environment upon MSG exposure. qPCR was performed to quantify the change in mRNA expression of MMP-2, MMP-9, TIMP-1, TIMP-2, in the HTR-8/SVneo trophoblast cells and SYN -1, SYN-2, β-hCG and GCM1, in the BeWo trophoblast cells treated with 25 mM and 50 mM MSG for 24h. Statistical analysis was done by comparing the fold change in the 25mM and 50 mM MSG treated group with respect to the Control. The results were analysed by the $2^{-\triangle\triangle C.T.}$ method. Results showed no significant changes in trophoblast invasion and differentiation markers upon MSG treatment.

Table S1. Details of primers used in this study.
